# Emergence of SARS‐CoV‐2 spike protein at the vaccination site

**DOI:** 10.1002/iid3.827

**Published:** 2023-03-29

**Authors:** Annika Beck, Hanna Dietenberger, Sebastian N. Kunz, Kevin Mellert, Peter Möller

**Affiliations:** ^1^ Institute of Pathology University Hospital of Ulm Ulm Germany; ^2^ Institute of Forensic Medicine University Ulm Ulm Germany

**Keywords:** COVID‐19, injection site, spike protein, vaccination

## Abstract

**Background:**

The anti‐coronavirus disease 2019 (COVID‐19) vaccines are of paramount importance in the fight against the COVID‐19 pandemic. Both viral vector‐ and nucleic acid‐based vaccines are known to effectively induce protection against the severe acute respiratory syndrome coronavirus 2 (SARS‐CoV‐2) virus by generating high antibody titers and effective T‐cell responses to the spike protein they encode. Although these vaccines are being applied worldwide and have been extensively investigated, the immunomorphological events at the vaccination site with respect to SARS‐CoV‐2 spike protein expression have not yet been described.

**Methods:**

We had the opportunity to examine the deltoid muscles of three men who died shortly after vaccination for unrelated reasons. We examined the vaccination sites histologically and immunohistochemically with various antibodies. Furthermore we incubated two different cell lines with one vaccine and examined the expression of the spike protein.

**Results:**

The vaccination sites show a dense lymphohistiocytic interstitial infiltrate which surrounds the small vessels and extends into the perimysium. The spike protein is expressed by histiocytic cells with a dendritic shape that are CD68‐positive and CD207‐negative, fibrocytes, and very rare S100‐positive cells. Interestingly, the skeletal muscle, being constitutively human leukocyte antigen (HLA)‐A,B,C‐negative, is induced at different levels in each specimen. In a cell culture experiment, we confirmed the ability of fibroblasts and interdigitating dendritic sarcoma cells to express spike protein in vitro after incubation with the Comirnaty vaccine.

**Conclusions:**

Histiocytic cells and fibrocytes are the heralds of spike protein synthesis at the vaccination site. The underlying cause of this apparent cell specifity is unknown. This needs to be investigated in future experiments, for example in an animal model.

## Graphical abstract

Investigation of the morphological changes at the anti‐severe acute respiratory syndrome coronavirus 2 (SARS‐CoV‐2) vaccination site reveals an inflammatory infiltrate. We provide a characterization of this infiltrate by immunohistochemistry and demonstrate the expression of the spike protein.

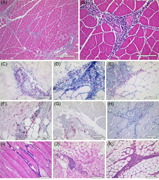

## 1 INTRODUCTION

The different authorized and approved vaccines are effective in inducing high titers of antibodies against the severe acute respiratory syndrome coronavirus 2 (SARS‐CoV‐2) spike protein.^1^, ^2^

The injection sites of different vaccines have been examined thoroughly with detailed information regarding gene expression, changes in microenvironment, and cellular composition in mice and humans.^3^, ^4^, ^5^, ^6^, ^7^ Furthermore, early events after the injection of an influenza vaccine in an explanted human skin model have been studied.^8^ Morphological animal studies have shown the correlation between histology and magnetic resonance imaging, with the aim of reducing the number of animals needed to be killed during the development of new veterinary vaccines.^9^ However, there are no publications on detailed immunomorphological studies of the injection site in human skeletal muscles.

We report three cases of early sudden deaths 2–4 days after vaccination with different coronavirus disease (COVID) vaccines. Two autopsies were performed in the Department of Pathology at the University Hospital Ulm, Germany (cases 1 and 2). The other postmortem was done at the Institute of Forensic Medicine in Ulm in collaboration with the Department of Pathology at Ulm University Hospital (case 3). Besides routine examination, a thorough sampling of different tissues for bio‐repository was done in all three cases. This was carried out within the scope of the project “COVID‐19 Autopsie‐ und Bioproben‐Register BW.” The next‐o‐kin gave their informed consent to the storage and usage of the tissues in this scientific context. The study was in accordance with the Declaration of Helsinki.

The workup of each of the three cases, including anamnestic data and histomorphological examination, led to the conclusion that death had occurred without any relationship to the vaccination. The stated causes of death were sudden cardiac death in a patient with severe myocardial hypertrophy, acute cardiac failure in a multimorbid patient, and aspiration of gastric content in a drug addict, respectively.

### 1.1 Case history

The first case (case 1) was a 61‐year‐old male. He had received his first injection of Vaxzevria (AstraZeneca) 3 days before death. Relatives reported a sudden onset of dyspnea followed by collapse. The emergency doctor detected severe bradycardia and was unable to re‐establish sufficient cardiocirculatory function. The autopsy revealed excessive left heart myocardial hypertrophy, severe coronary disease, and old myocardial scars due to previous infarctions. Sudden heart failure was regarded as the cause of death.

Case 2 was a 59‐year‐old male resident of a nursing home. He was physically disabled due to multisystem atrophy and had received his first dose of COVID‐19 Vaccine Janssen (Johnson & Johnson). Four days after the injection he developed a severe disturbance of consciousness including confusion. Shortly afterwards he was found dead. Besides his neurological disorder, he had suffered from hypertension, type 2 diabetes, pulmonary emphysema, and general atherosclerosis. The autopsy did not reveal a clear cause of death. In accordance with his medical history, acute cardiac failure due to arrythmia was regarded as the most likely cause.

Case 3 was a 20‐year‐old male drug addict who had received his first injection of Comirnaty (BioNTech/Pfizer) 2 days before his unexpected death. The known drug abuser, who was found dead in his home, died of asphyxia by aspirating gastric content due to intoxication.

## 2 MATERIAL AND METHODS

The deltoid muscles (*n* = 3) were obtained during autopsy and fixed in formalin for approximately 72 h. Thereafter, the entire muscle was examined by routine histology to localize the injection site. Once identified, this small area was chosen for serial sectioning for immunohistochemistry.

Immunohistochemistry was performed on 2–3 µm thick sections of formalin‐fixed paraffin‐embedded tissue on DAKO Autostainer PLUS (Agilent). Antigen retrieval was performed by microwave heating in citrate buffer, pH6, for 20 min. The sections were incubated for 1 h with a panel of monoclonal antibodies. These were anti‐human leukicyte antigen (HLA)‐A,B,C (clone EMR8‐5, Abcam), anti‐HLA‐DR (clone 1B5, Agilent), CD8 (clone C8/144B, Agilent), CD4 (clone 4B12, Agilent), CD20 (clone L26, Agilent), CD138 (clone MI15, Agilent), CD68 (clone PG‐M1, Agilent), CD207 (Cell Marque), and anti‐COVID‐19 spike protein (clone HL257, Abcam). We also applied a polyclonal anti‐CD3 antibody (GA503, Agilent) and a polyclonal anti‐S100 protein (GA504, Agilent). Bound antibodies were visualized by the DAKO REAL Detection System (alkaline phosphatase/RED/Rabbit/Mouse, K5005, Agilent) and counterstained with hematoxylin following standard protocols. All antibodies are used in our diagnostic routine and therefore positive and negative controls are performed regularly.

Additionally, we performed immunostainings with the anti‐spike‐antibody on the myocard of the left ventricles of each deceased person according to above‐mentioned protocol and yielded negative results (Supporting Information: Figure 13).

Microscopic photographs were taken with a photo microscope (Axiophot) with a charge‐coupled device (CCD) camera (JVC, KY‐F75U) and the software Diskus (Hilgers Technisches Büro).

The interdigitating dendritic sarcoma cell line U‐DCS and primary foreskin fibroblasts of two different donors were obtained from the cell bank of the Institute of Pathology, University Hospital Ulm. The experiments were in line with the Declaration of Helsinki, and the patient and donors gave their written consent for the scientific use of the donated tissue. The cells were seeded in densities of 3000 cells per cm² in eight well µ‐slides (Ibidi). For incubation of the cells, a dose of Comirnaty vaccine was prepared as recommended for vaccination. The vaccine was diluted 1:100 in cell culture media, and 300 µl of the mixture was applied to the cell culture wells. Nontreated cells served as a negative background staining control. All experiments were performed as technical triplicates (*n* = 3). After the respective incubation time, the cells were fixed for 10 min at −20°C in 3:1 mixed methanol: glacial acetic acid and washed twice in phosphate‐buffered saline. Detection of the spike protein was performed following standard protocols for immunocytochemistry using a recombinant Anti‐SARS‐CoV‐2 Spike Glycoprotein S1 antibody (Abcam, ab281306, clone HL257, dilution 1:100) and the DAKO REAL^TM^ Detection System Alkaline Phosphatase/RED Rabbit/Mouse (DAKO). Pictures of the cells were taken using a Leica DM IL inverted microscope (Leica Camera AG).

## 3 RESULTS

The three men died 3 (case 1), 4 (case 2), and 2 days (case 3) after having received their first COVID‐19 vaccination, that is, at a time when the deltoid muscle is often still painful at the injection site.

### 3.1 Features common to the three injection sites

**Figure 1:**
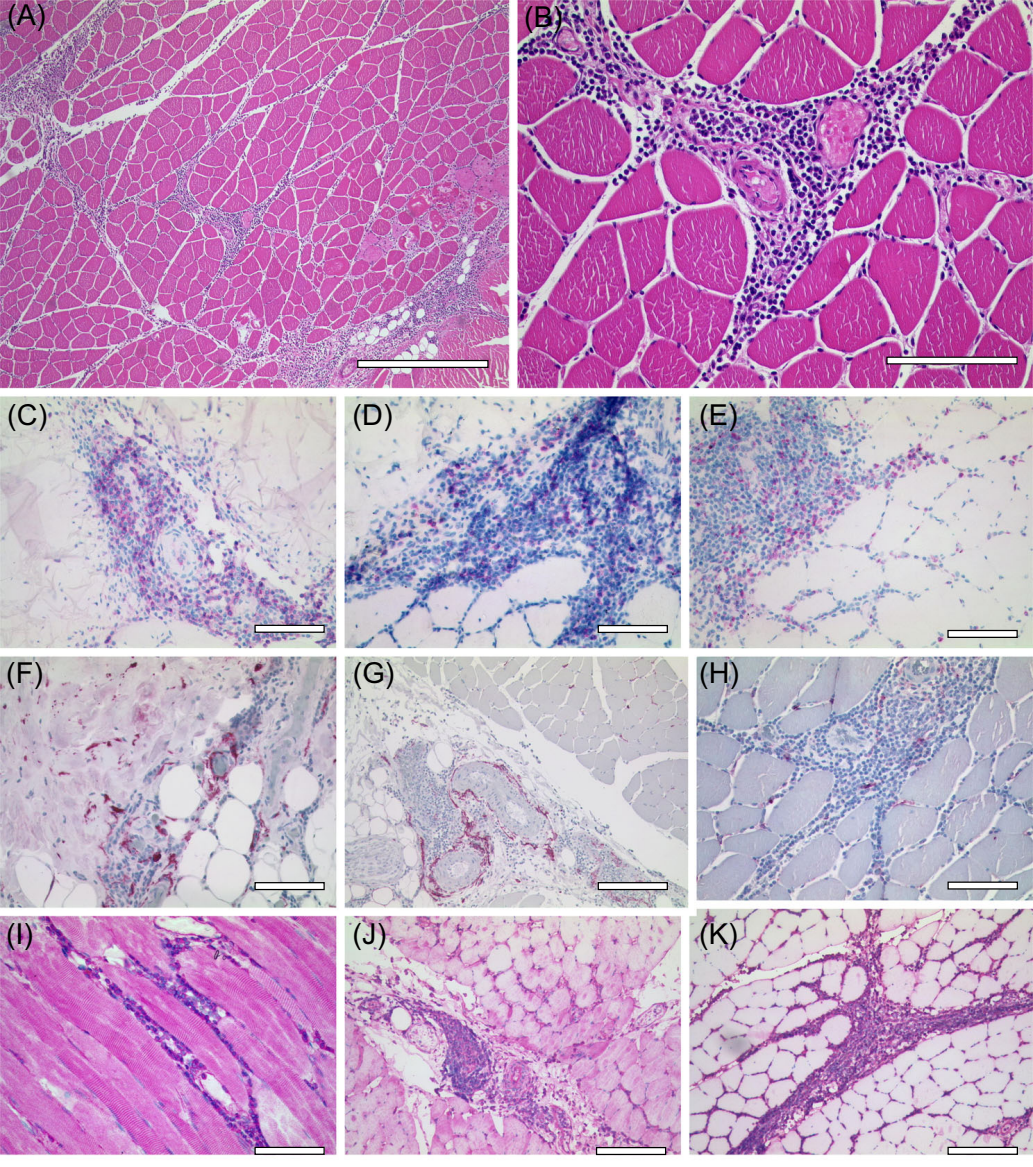
Histology of the vaccination sites (*n* = 3). (A, B) The injection site of Comirnaty (case 3), 2 days after vaccination, shows a lymphohistiocytic infiltrate ((A) HE, scale bar 500 µm) with a prominent perivascular distribution ((B) HE, scale bar 100 µm). (C, E) Cellular composition of the reactive infiltrate of case 3: At the Comirnaty injection site, there are more CD4‐positive T cells ((C) CD4, scale bar 100 µm, CD4) than CD8‐positive T cells ((D) CD8, scale bar 100 µm) and macrophages ((E) CD68, scale bar 100 µm) (F, H) Comparison of the spike protein expression in all three cases: The spike protein is expressed in numerous histiocytes and fibrocytes at the injection site of Vaxzevria (case 1) ((F) spike, scale bar 100 µm) and COVID‐19 Vaccine Janssen (Case 2) ((G) spike, scale bar 200 µm). These spike protein‐expressing cells were less numerous at the injection site of Comirnaty (case 3) ((H) spike, scale bar 100 µm). (I, K) HLA‐A,B,C was heavily induced at the injection site of Vaxzevria (case 1) ((I) HLA‐A,B,C, scale bar 100 µm), mildly induced at the injection site of COVID‐19 Vaccine Janssen (case 2) ((J) HLA‐A,B,C, scale bar 200 µm) and not induced at the injection site of Comirnaty (case 3) ((K) HLA‐A,B,C, scale bar 200 µm). All stains were performed once per sample and due to the small sample size, the comparison was done solely on a visual basis. No statistic test was applicable.

At the injection site, a localized lymphohistiocytic infiltrate of 1–2 cm in maximal diameter had formed around small blood vessels (Figure 1 and Supporting Information: Figure 1). These infiltrates extended into the skeletal muscle along thin septa in the muscular interstitium.

The lymphocytes were almost exclusively T cells visualized by CD3 staining (Supporting Information: Figure 2). CD20‐positive B cells made up approximately 1% (Supporting Information: Figure 3). CD138‐positive plasma cells were virtually absent (Supporting Information: Figure 4). Not one single granulocyte was detected.

The second most frequent cell type was the CD68‐positive macrophage, which accounted for approximately 40% of the mononuclear cells (Supporting Information: Figure 5). These CD68‐positive cells were more mobile than the T cells. In contrast to the T cells, which were located mainly at perivascular sites, the macrophages made up the majority of cells infiltrating the muscular interstitial spaces. S100 protein‐expressing histiocytic cells were scarce, comprising less than 1% of the infiltrate, and were located amidst the lymphocyte‐dominated perivascular spaces, but also and very infrequently among muscle cells (Supporting Information: Figure 6). CD207‐expressing Langerhans cells were not detected (Supporting Information: Figure 7). As a key feature, the spike protein was detected at a high intracytoplasmic concentration in histiocytes and fibrocytes.

HLA‐DR was present in a major subset of the lymphohistiocytic infiltrate and in interstitial dendritic cells within the muscular interstitium, while the entire skeletal muscle, local nerve fibers, and blood vessels were HLA‐DR‐negative (Supporting Information: Figure 8).

### 3.2 Features differing among the three vaccination sites

The most impressive difference was found for HLA‐A,B,C expression (Supporting Information: Figure 9). Under normal conditions, skeletal muscle cells, that is, distant to the injection site were completely devoid of class‐I histocompatibility complex proteins. By contrast, all other cells, vascular endothelial cells, interstitial cells, and all cells of the mobile infiltrate were strongly HLA‐A,B,C positive. The skeletal muscle cells at the site of the Vaxzevria (AstraZeneca) injection (case 1) were heavily induced for HLA‐A,B,C. This effect faded with the distance to the inflammatory epicenter. At the site where COVID‐19 Vaccine Janssen (Johnson & Johnson) had been administered (case 2), this also occurred, but to a far lesser extent. However, at the site where Comirnaty (BioNTech/Pfizer) had been applied (case 3) the skeletal muscle cells remained HLA‐A,B,C‐negative.

There were minor differences in the amount of spike protein‐expressing dendritic cells and fibrocytes (Supporting Information: Figure 10). They were likewise numerous in the Vaxzevria (AstraZeneca) (case1) and COVID‐19 Vaccine Janssen (Johnson & Johnson) (case 2) induced inflammation but somewhat less frequent in the reaction induced by Comirnaty (BioNTech/Pfizer) (case 3).

Furthermore, we observed minor differences in the CD4 and CD8 subsets at the vaccination sites (Supporting Information: Figures 11 and 12): The Vaxzevria (AstraZeneca)‐induced infiltrate (case 1) contained about three times more CD8‐positive cells than CD4‐positive cells. The COVID‐19 Vaccine Janssen (Johnson & Johnson)‐induced inflammation (case 2) contained more CD4‐ than CD8‐positive cells, while CD4‐positive cells predominated in the infiltrate evoked by Comirnaty (BioNTech/Pfizer) (case 3).

**Figure 2:**
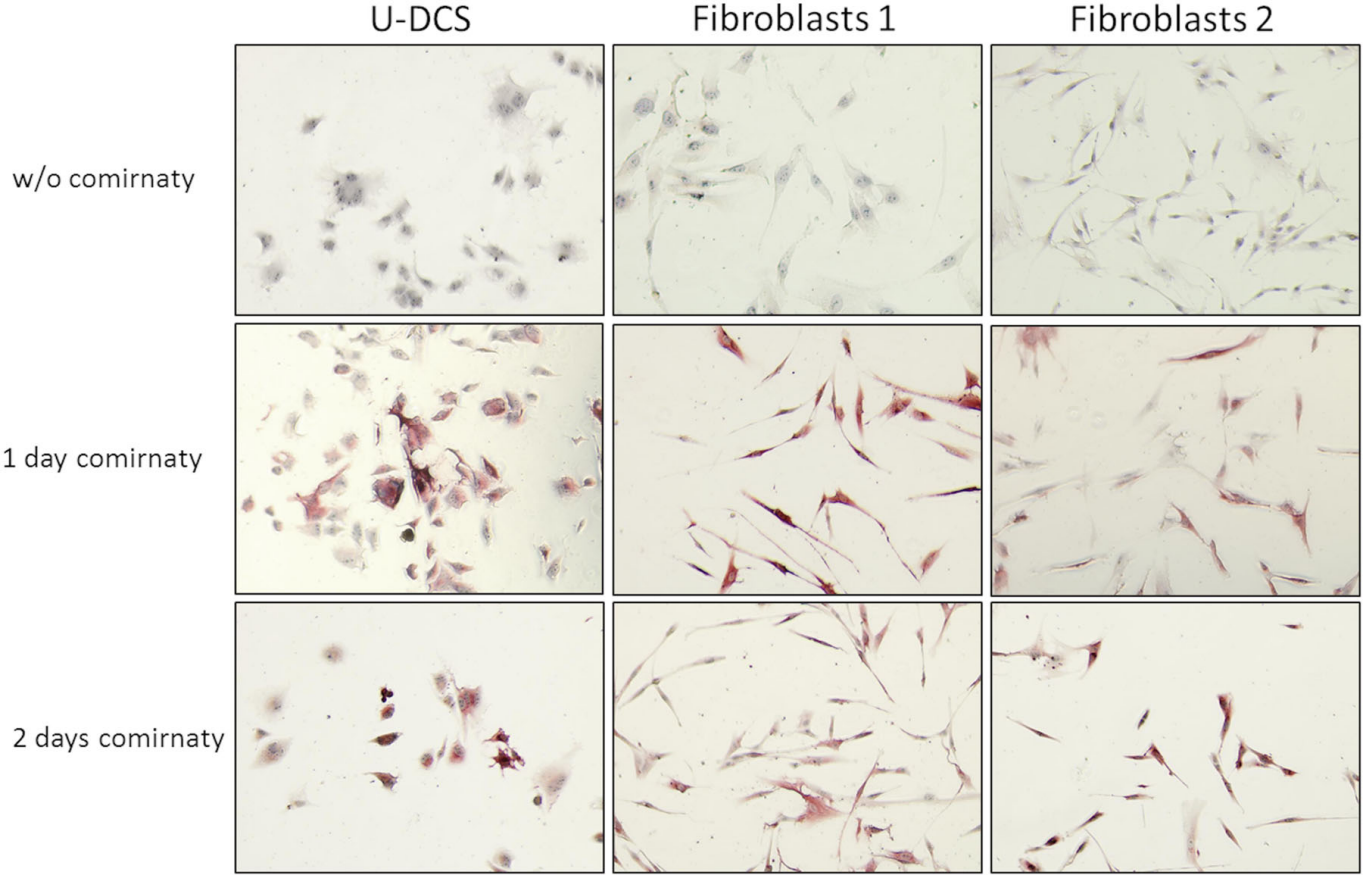
Immunocytological detection of the spike protein. The interdigitating dendritic sarcoma cell line U‐DCS and the fibroblasts of two healthy donors were incubated for 1 or 2 days with the Comirnaty vaccine in vitro. Cells without treatment (w/o Comirnaty) served as a control for background staining. Spike proteins are visualized by the color red. All the cell types tested show clear positivity after both incubation times. Experiments were performed as technical triplicates.

The in vitro incubation of the interdigitating dendritic sarcoma cells (U‐DCS) and fibroblasts of two different donors resulted in clearly detectable amounts of spike protein within the cytoplasm of the cells. Positivity persisted in both cell types for at least 48 h (Figure 2).

## 4 DISCUSSION

We provide compelling evidence and are the first to show that within the first few days after vaccination with messenger RNA (mRNA) or viral vector‐based vaccines encoding the COVID‐19 spike protein this immunogenic protein is exclusively synthesized in histiocytic cells with dendritic morphology and fibrocytes but not in other resident cells or lymphocytes.

All of our three cases showed a similar distribution of the spike protein in the examined tissue. Simian adenoviruses like ChAdOx1, which is the vector of Vaxzevria, are well known for their broad tissue tropism.^11^ For Ad26, the vector of COVID‐19 Vaccine Janssen, the cellular tropism is less clear. Integrins, sialic acid, and CD46 are discussed as entry receptors.^12^ The lipid nanoparticles (LNPs) of the Comirnaty vaccine, which allow the mRNA to enter the host cell, are not targeted.^10^ These facts pose the question of why can we see the expression of spike protein only in dendritic cells and fibrocytes.

The CD68‐positive cells in the background, however, clearly outnumbered the spike protein‐positive cells and are more roundish. It is conceivable that the advent of histiocytes in this micro area leads to spike protein production in a functional subset and takes some time. This can be hypothesized when considering that the person with less spike protein‐producing cells at his vaccination site, who had received Comirnaty (BioNTech/Pfizer) (case 3), died just 2 days after having been vaccinated.

It has been repeatedly shown that the induction of MHC molecules, for example, by interferon gamma, is a rapid process taking less than 48 h.^13^ The impressive differences in HLA‐A,B,C induction might therefore not only be due to differences in the time to observation, but might be explained by the composition of the vaccine. The minor differences in the CD4/CD8 ratios might also reflect differences in the local immune cellular interaction due to such differences. However, caution is advisable since what we have seen is based on single observations. Be it as it may it is unexpected that neither muscle cells nor lymphocytes nor endothelial cells expressed the spike protein but only histiocytic cells with dendritic morphology and fibrocytes do so. This pattern of cells expressing the spike protein was identical for all three vaccines independent of their composition or transfection strategy.

Against the background of experimental data on the uptake and fate of mRNA vaccines, this selectivity is remarkable since Shimosakai et al., for example, showed an uptake of mRNA‐loaded LNPs also in B‐lymphocytes.^14^

Our in vitro experiments showed that spike protein production of lipid‐shuttle‐based vaccine (Cormirnaty) treated cells can be detected shortly after vaccine exposure of cultured fibroblasts and an interdigitating dendritic sarcoma cell line. Considering that the maximal protein production after intradermal injection of mRNA in mice was reached after 24–48 h and that the estimated half‐life time for mRNA‐based vaccines is expected to be around 2 days^15^ the observed time span from vaccination until death (2–4 days after vaccination) in our study should have been sufficient for spike protein production in all cells that had taken up the spike protein‐coding nucleic acids. This enforces the hypothesis of a cell‐type restricted uptake of mRNA applied by Covid‐19 vaccines when being injected into the deltoid muscle. The underlying reason for this is by now unclear and needs to be invested systematically in mouse models.

## AUTHOR CONTRIBUTIONS

*Concept and design of study*: Peter Möller. *Performance of the autopsies in the Institute of Forensic Medicine*: Sebastian N. Kunz. *Performance of the autopsy in the Institute of Pathology*: Peter Möller, Hanna Dietenberger. *Evaluation of histology and immunohistochemistry*: Peter Möller, Annika Beck, Hanna Dietenberger. *Cell culture experiment*: Kevin Mellert. *Preparation of figures and writing of the manuscript*: Peter Möller, Annika Beck, Kevin Mellert. All authors read and agreed to the manuscript.

## CONFLICT OF INTEREST STATEMENT

The authors declare no conflict of interest.

## ETHICS STATEMENT

The study is in accordance with the Declaration of Helsinki. The next‐of‐kin family members gave their informed consent to the storage and usage of the tissues in this scientific context.

## Supporting information

Supporting information.

Supporting information.

Supporting information.

Supporting information.

Supporting information.

Supporting information.

Supporting information.

Supporting information.

Supporting information.

Supporting information.

Supporting information.

Supporting information.

Supporting information.

Supporting information.

## Data Availability

The data that support the findings of this study are available in the supplementary material of this article.
