# Supplementary figures and images for: Emergence of SARS‐CoV‐2 spike protein at the vaccination site
Source: Immun Inflamm Dis. 2023 Mar 29;11(3):e827. doi: 10.1002/iid3.827 (PMC10052447; doi:10.1002/iid3.827)

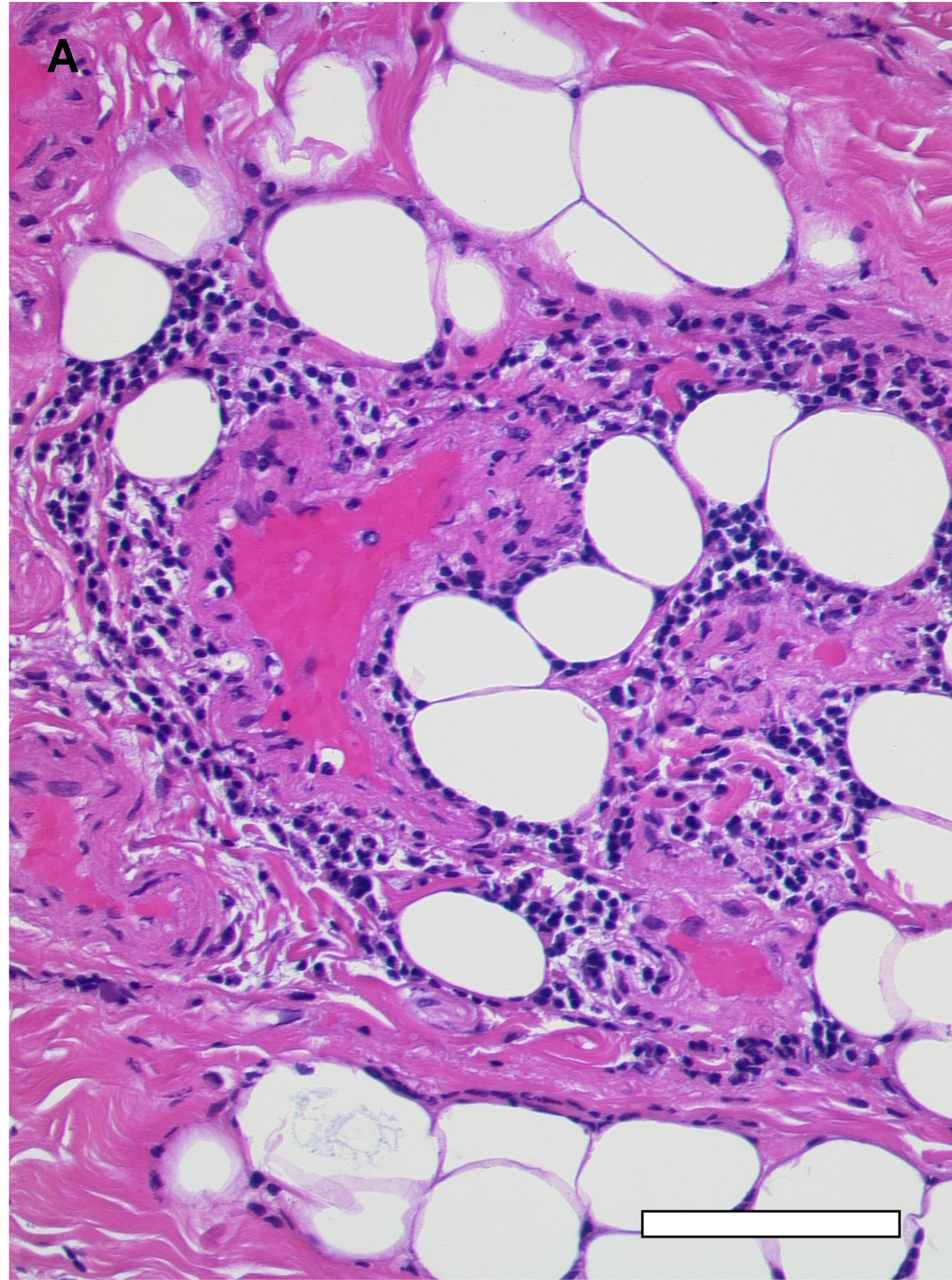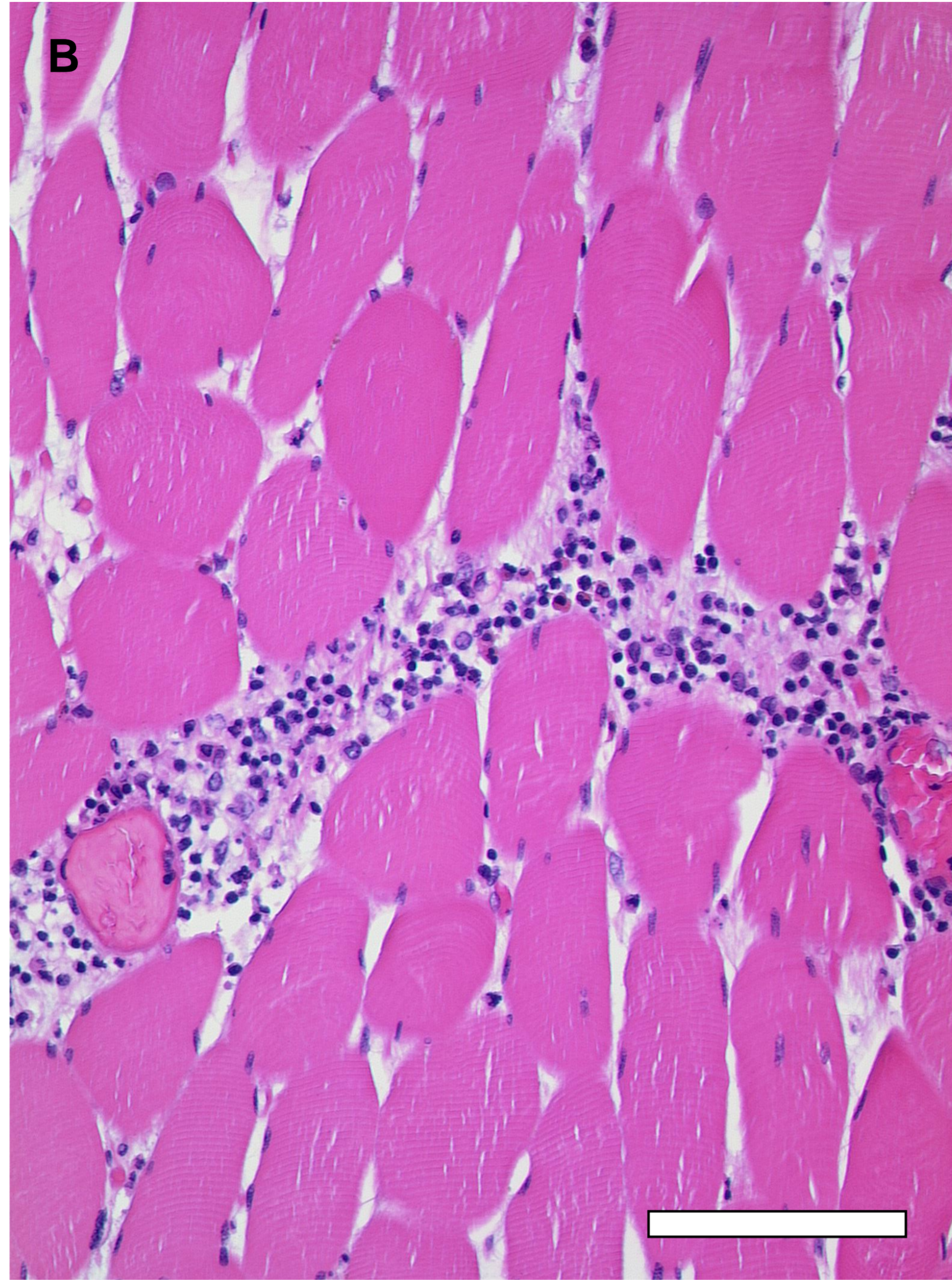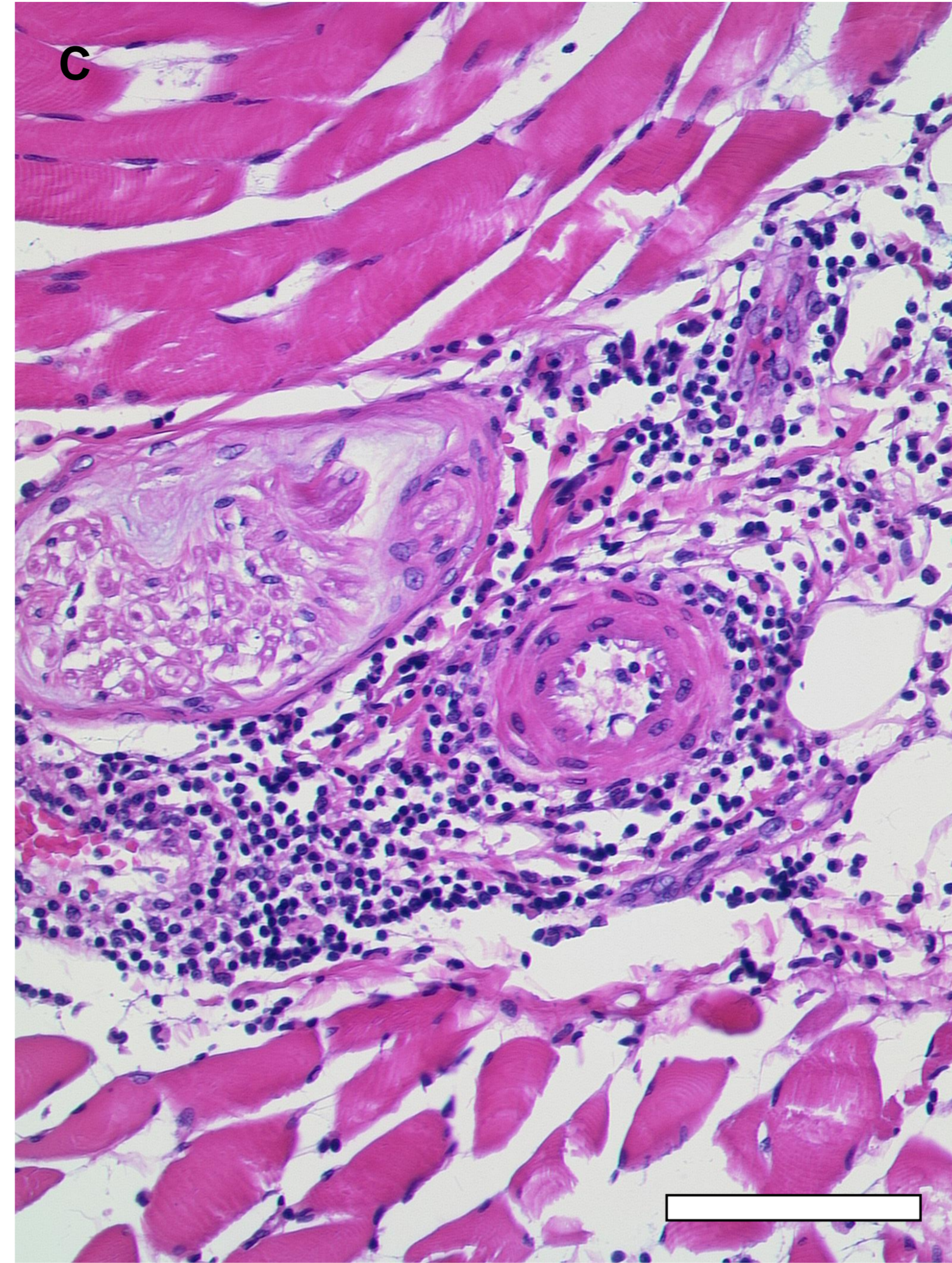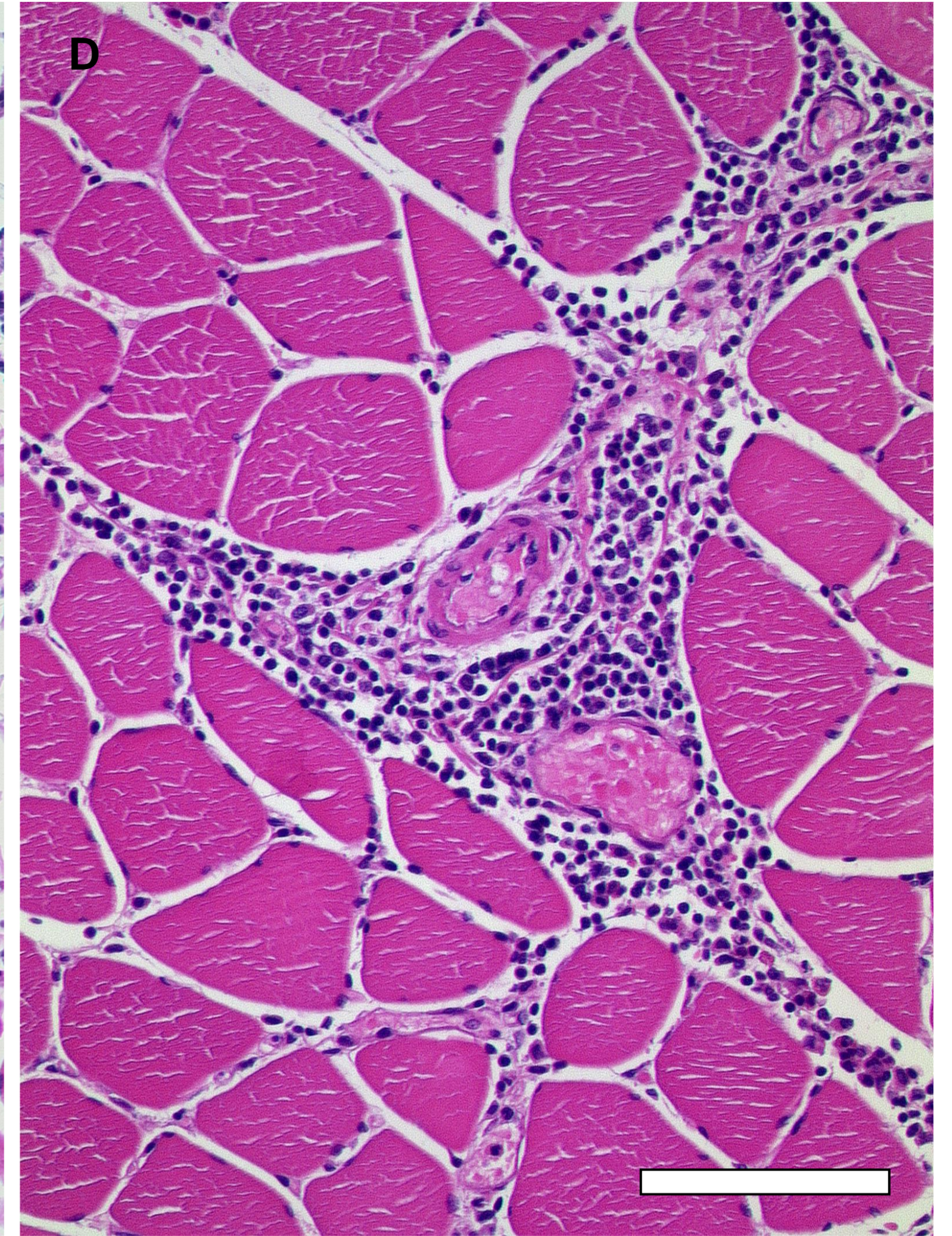

Supplement: Supplementary file 1 — Supporting information. [file IID3-11-e827-s011.pdf]

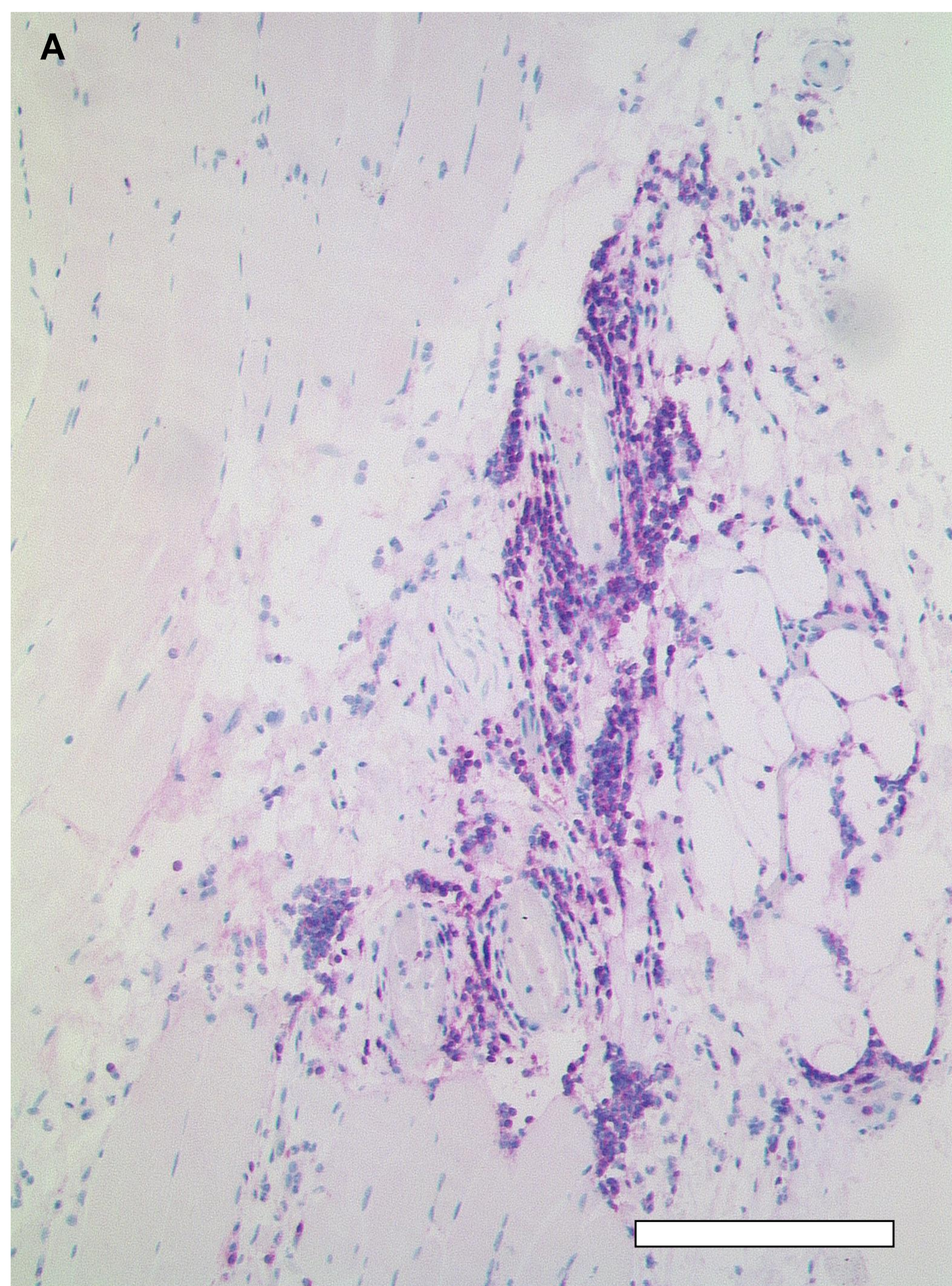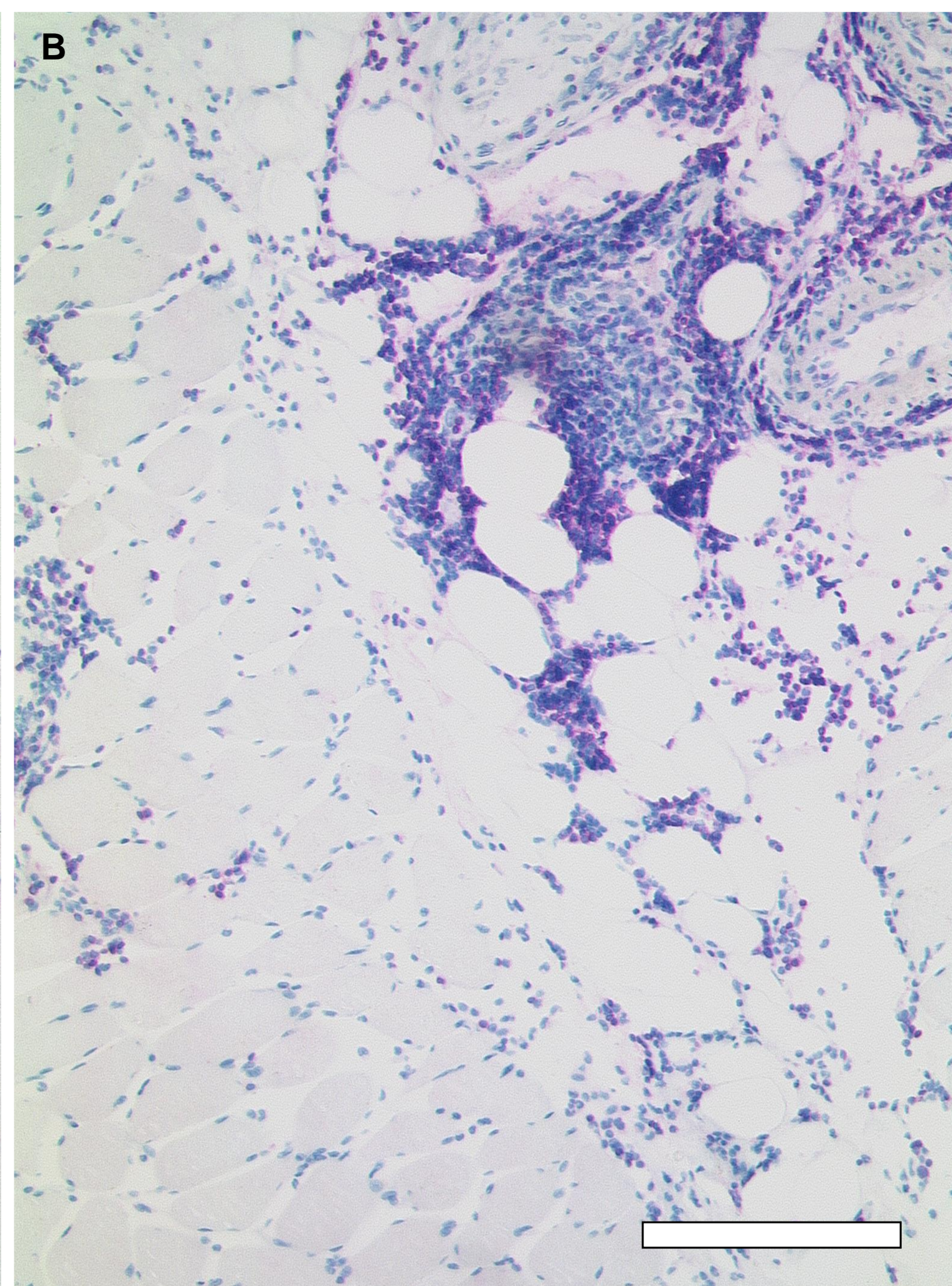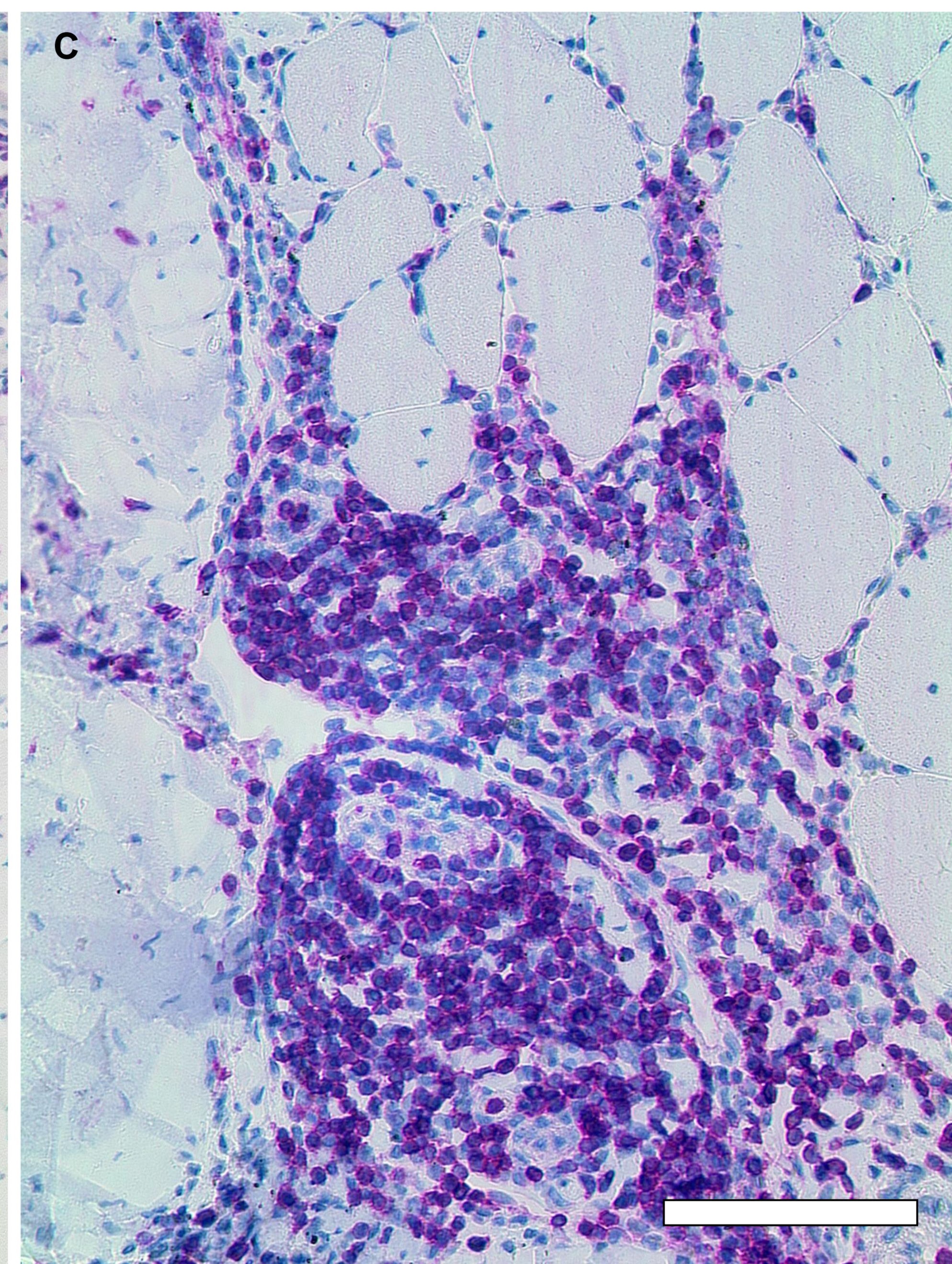

Supplement: Supplementary file 2 — Supporting information. [file IID3-11-e827-s006.pdf]

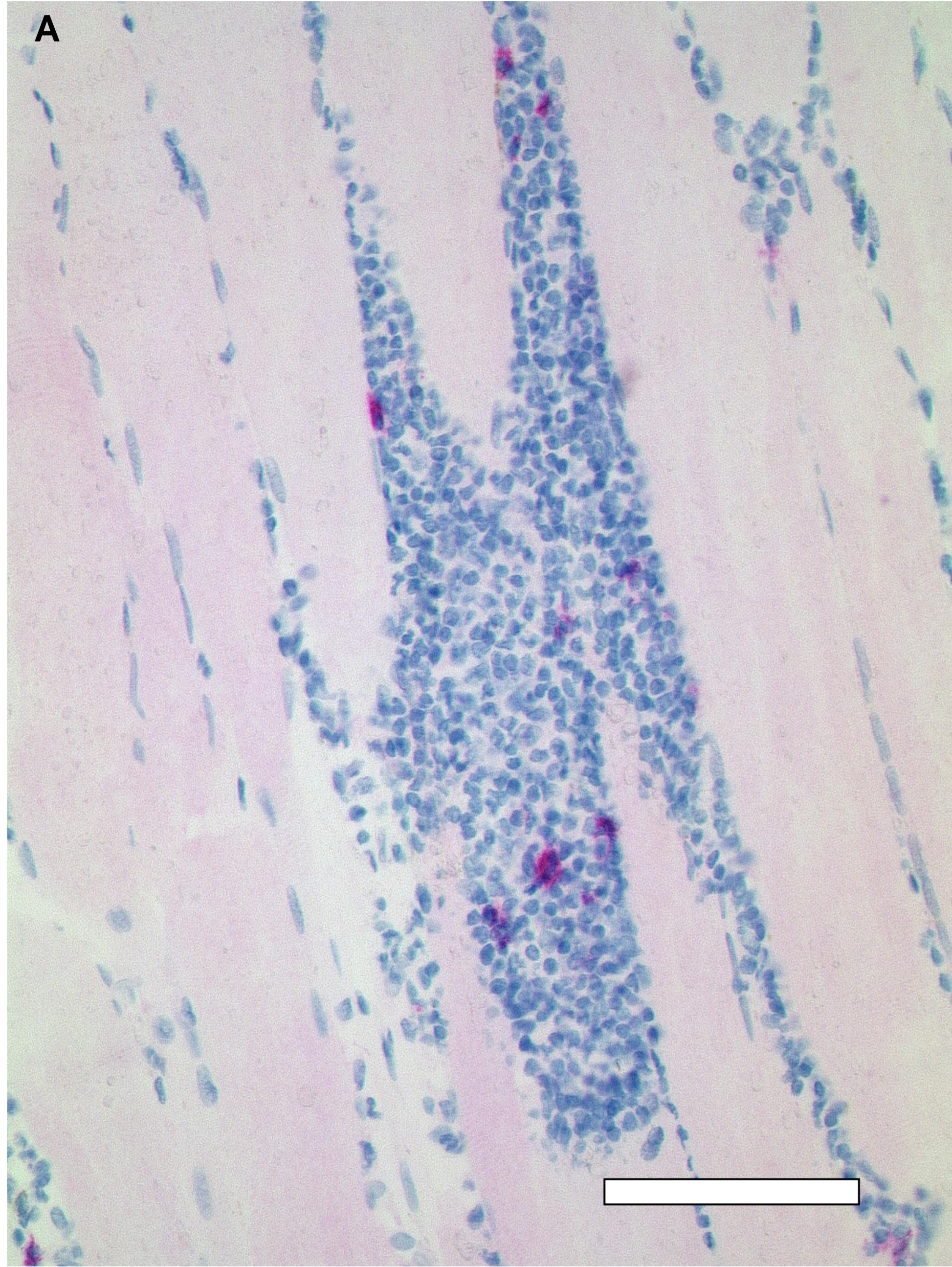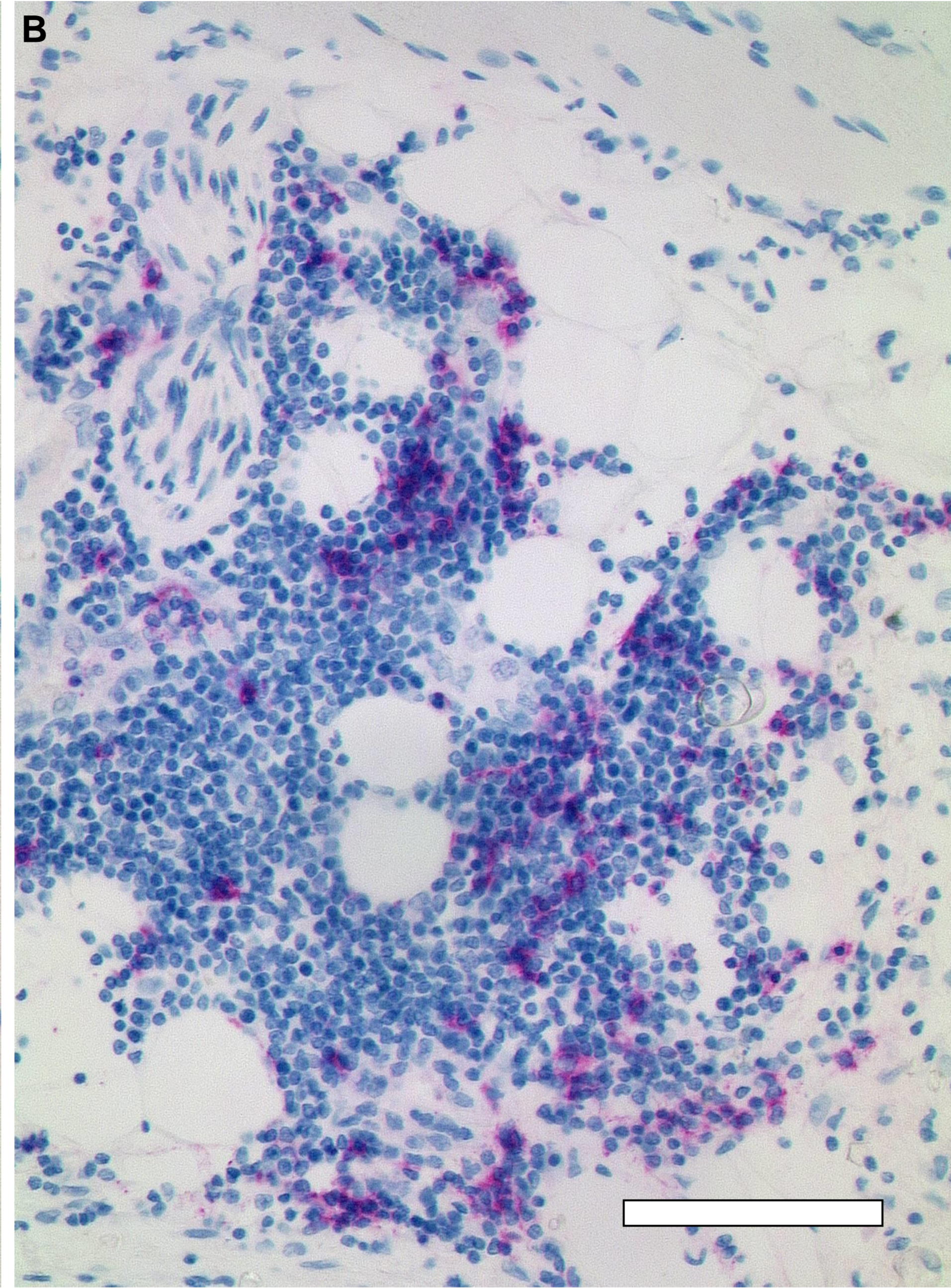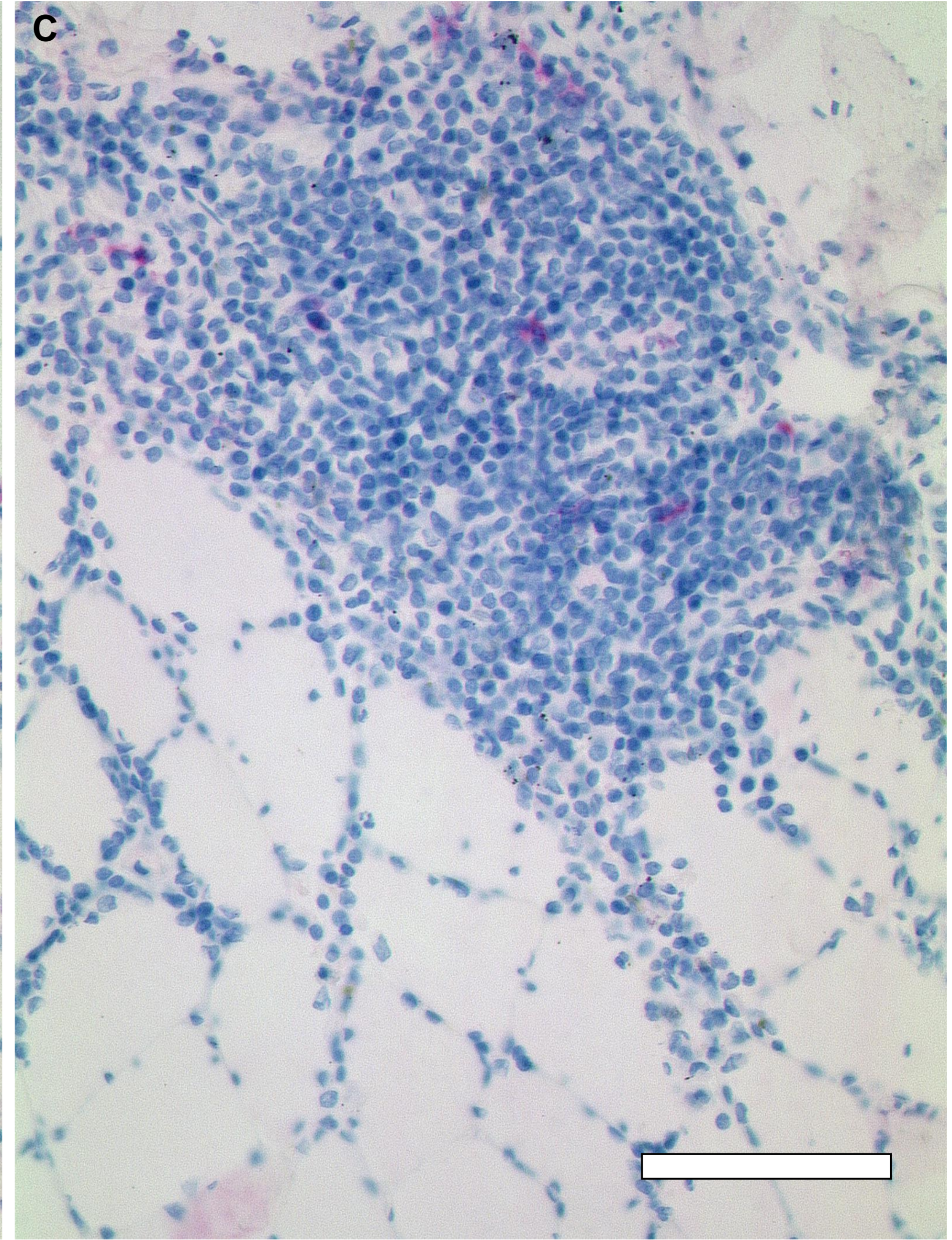

Supplement: Supplementary file 3 — Supporting information. [file IID3-11-e827-s001.pdf]

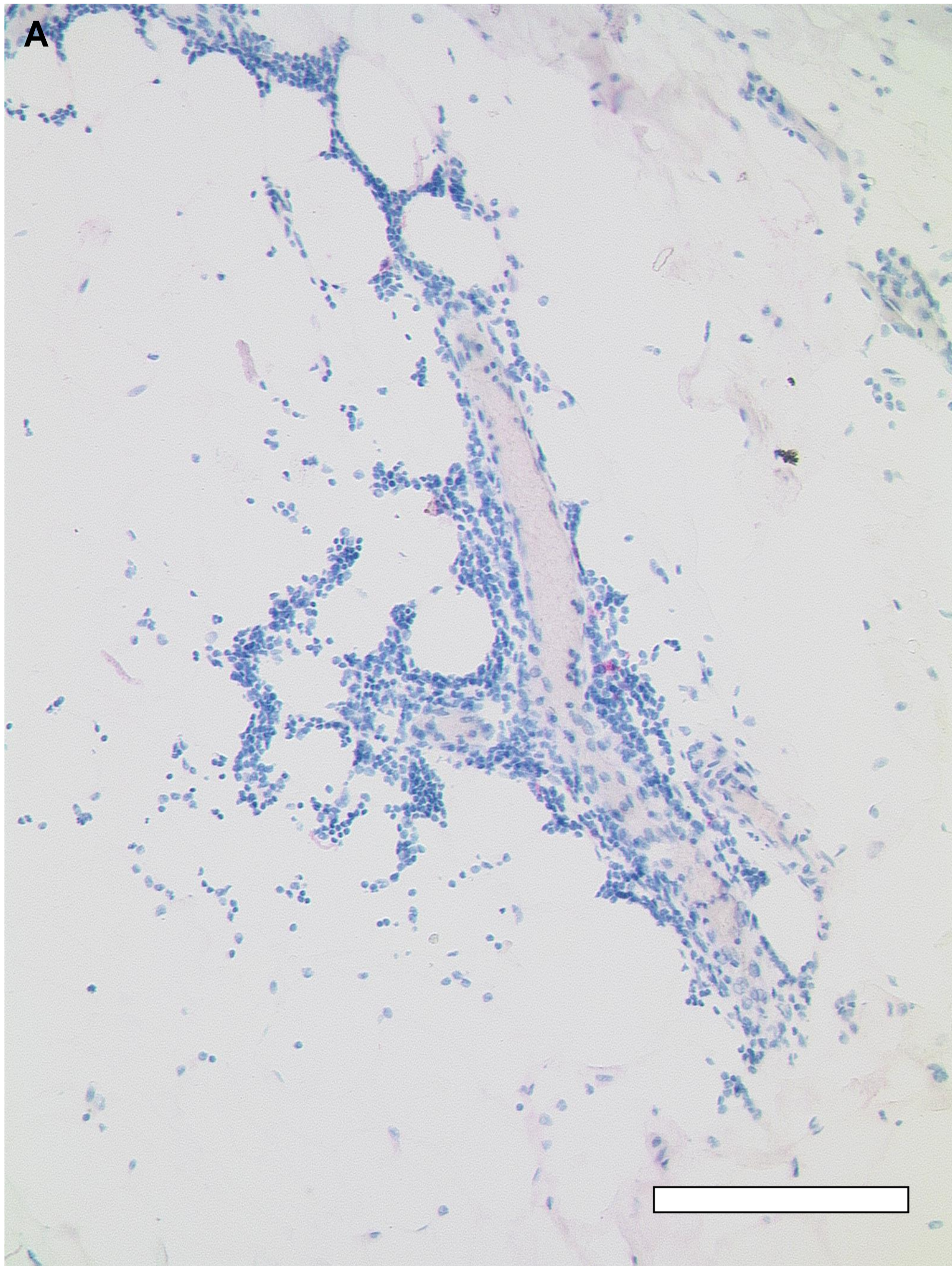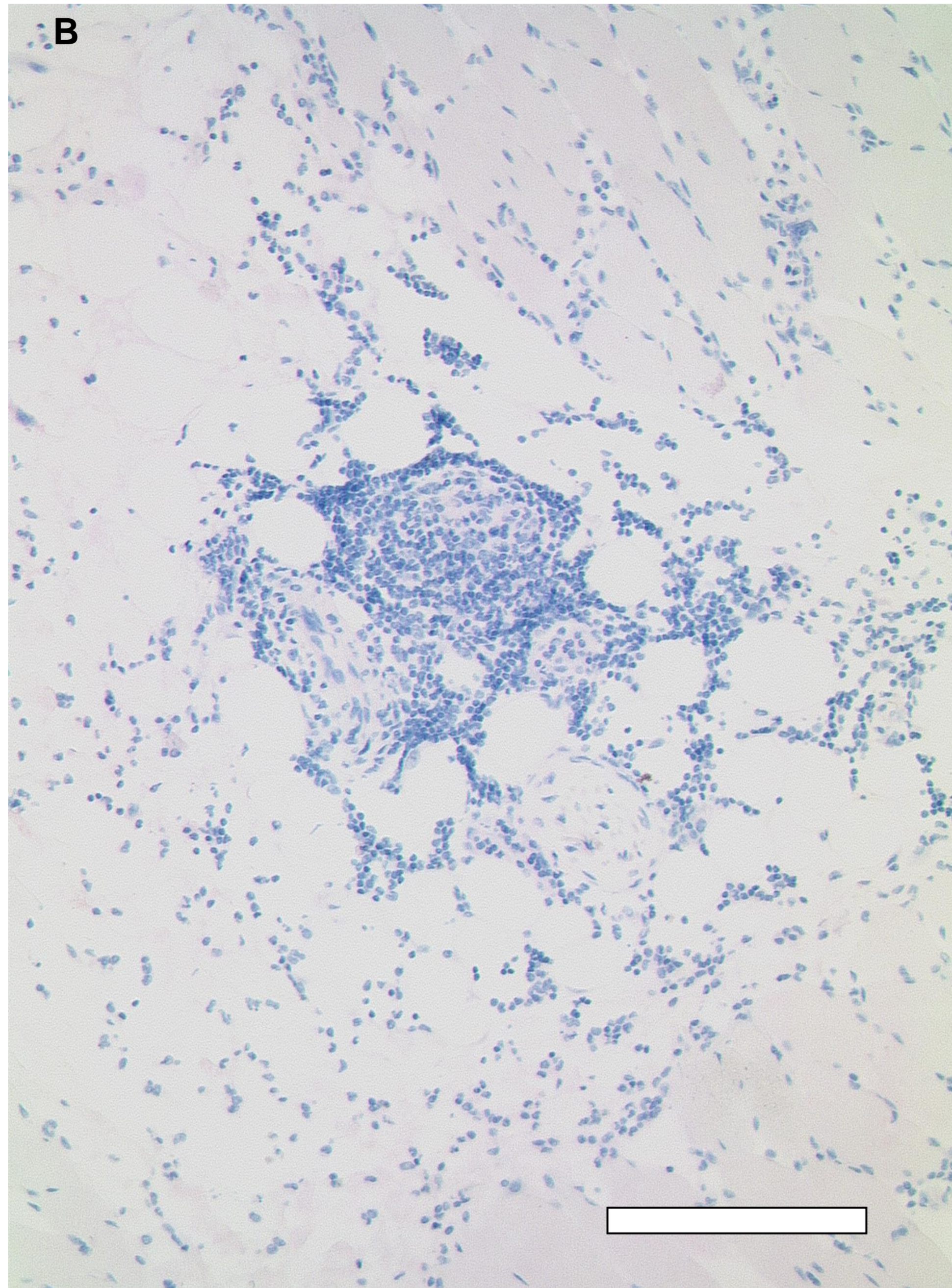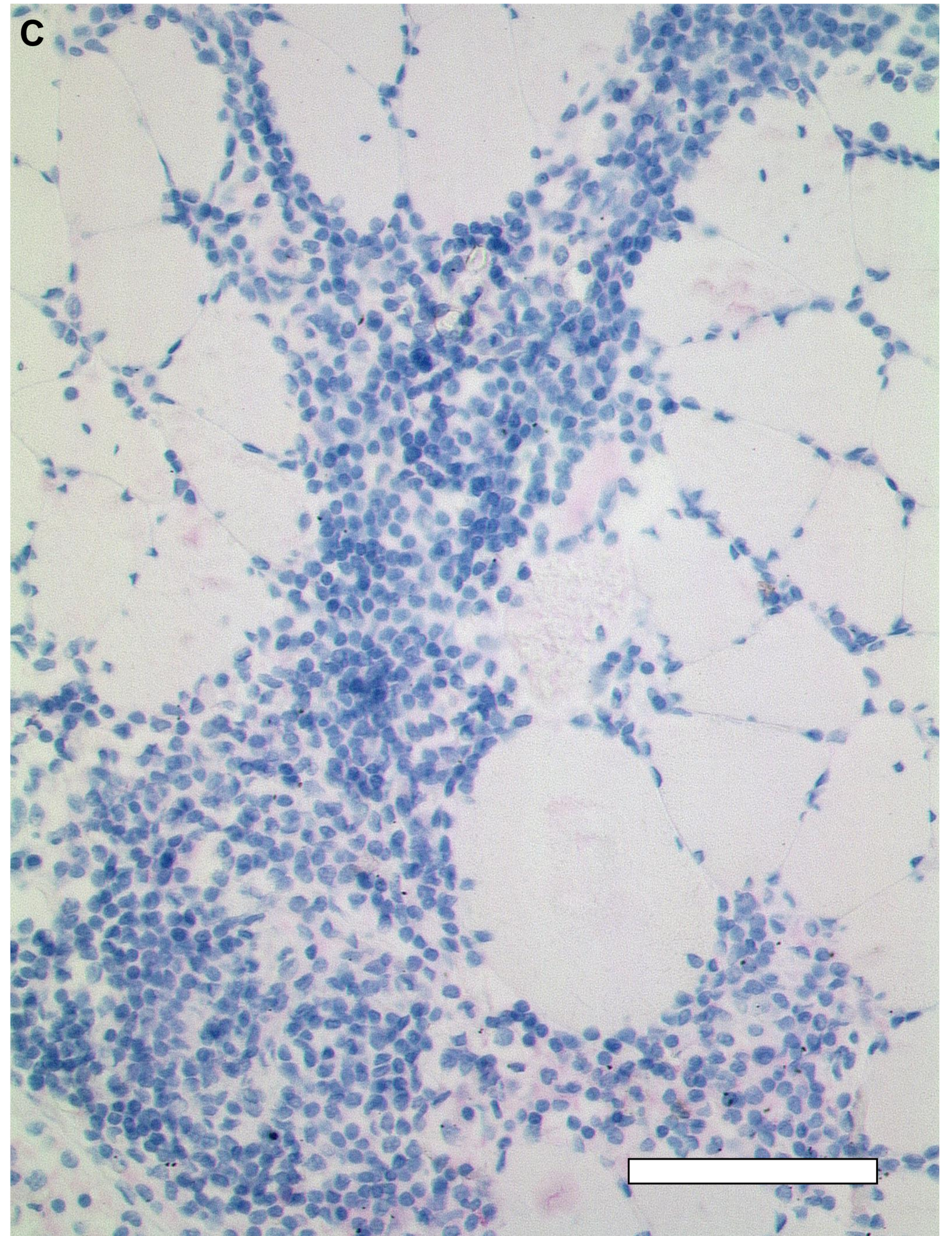

Supplement: Supplementary file 4 — Supporting information. [file IID3-11-e827-s008.pdf]

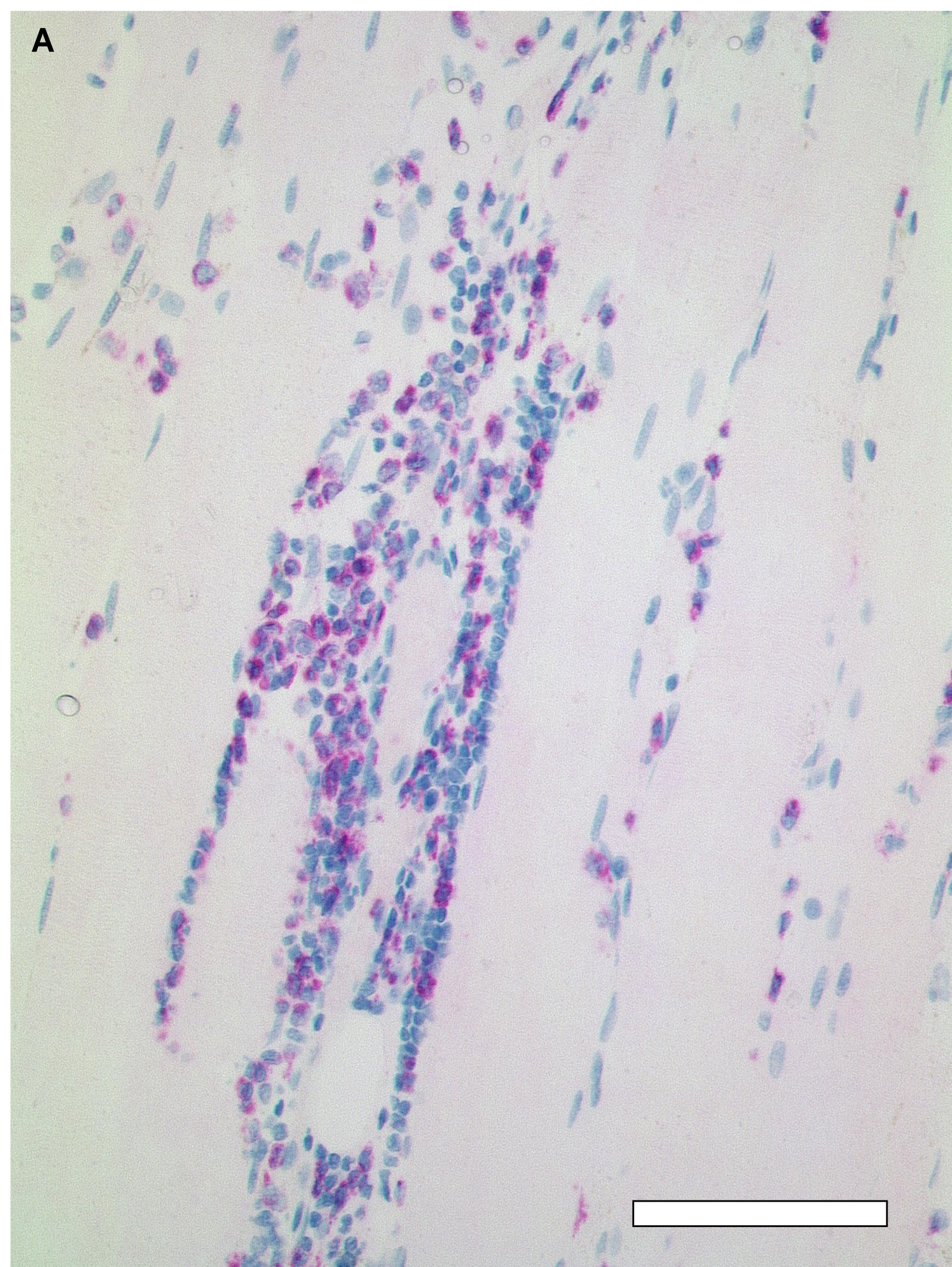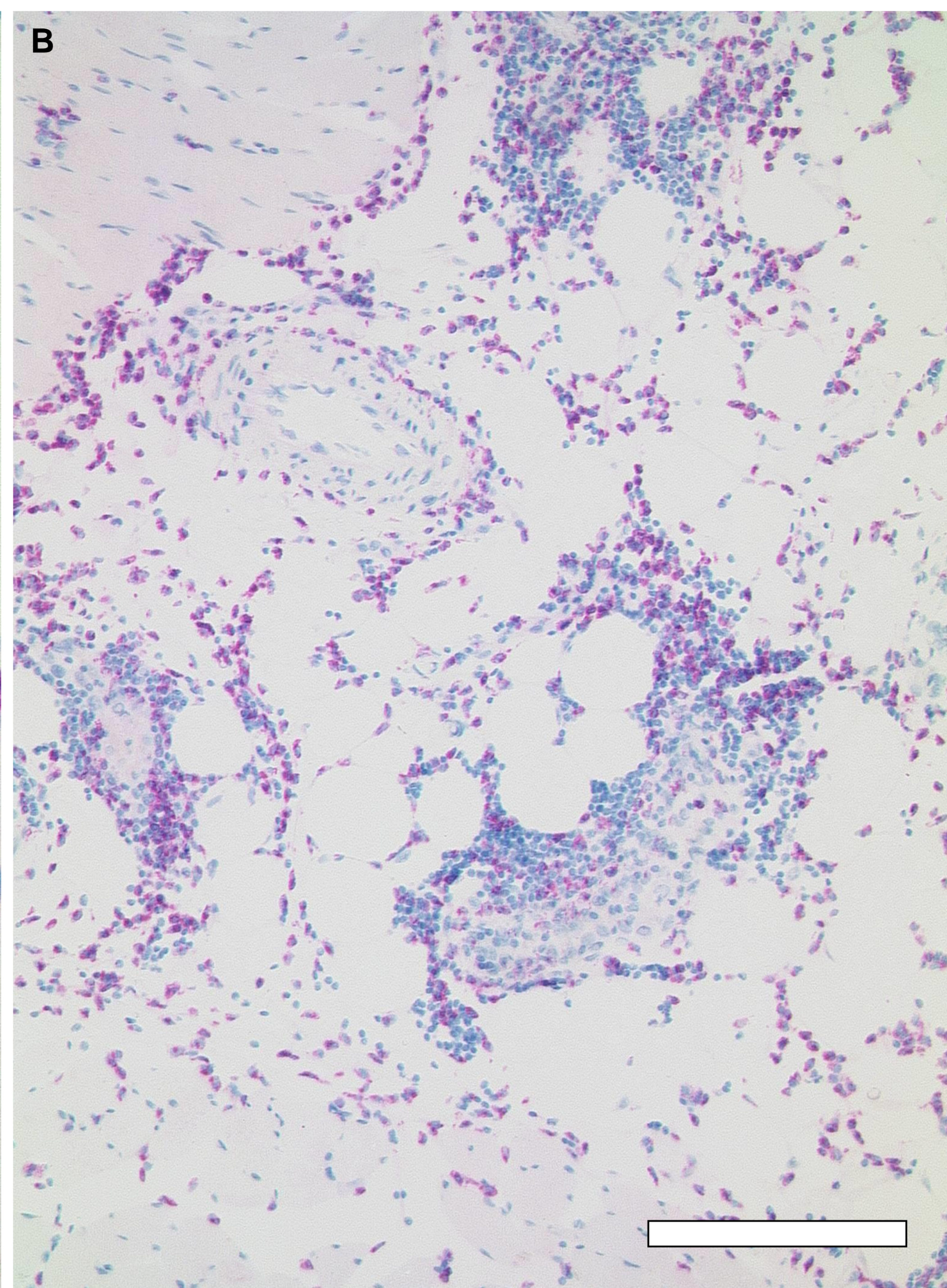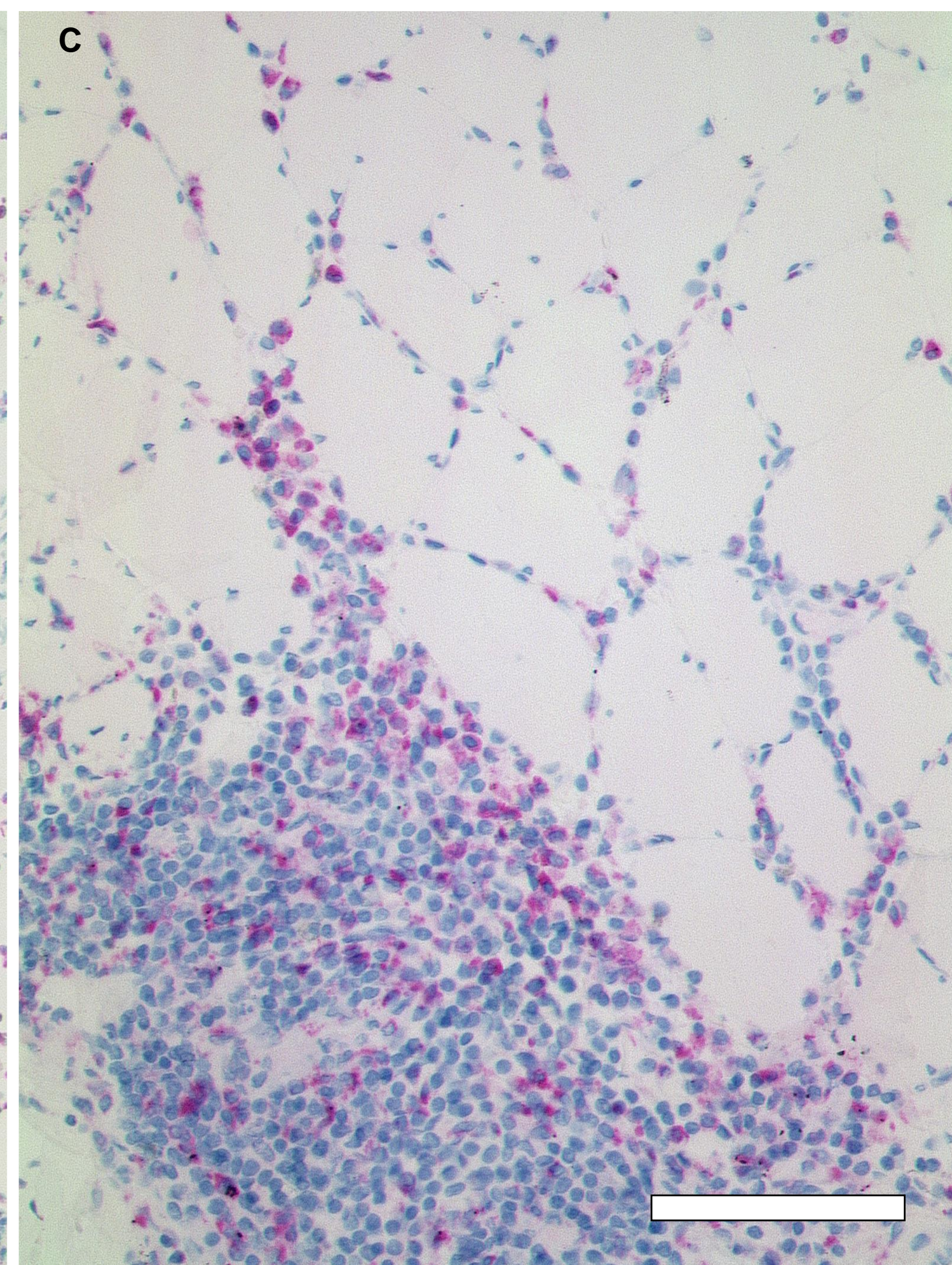

Supplement: Supplementary file 5 — Supporting information. [file IID3-11-e827-s003.pdf]

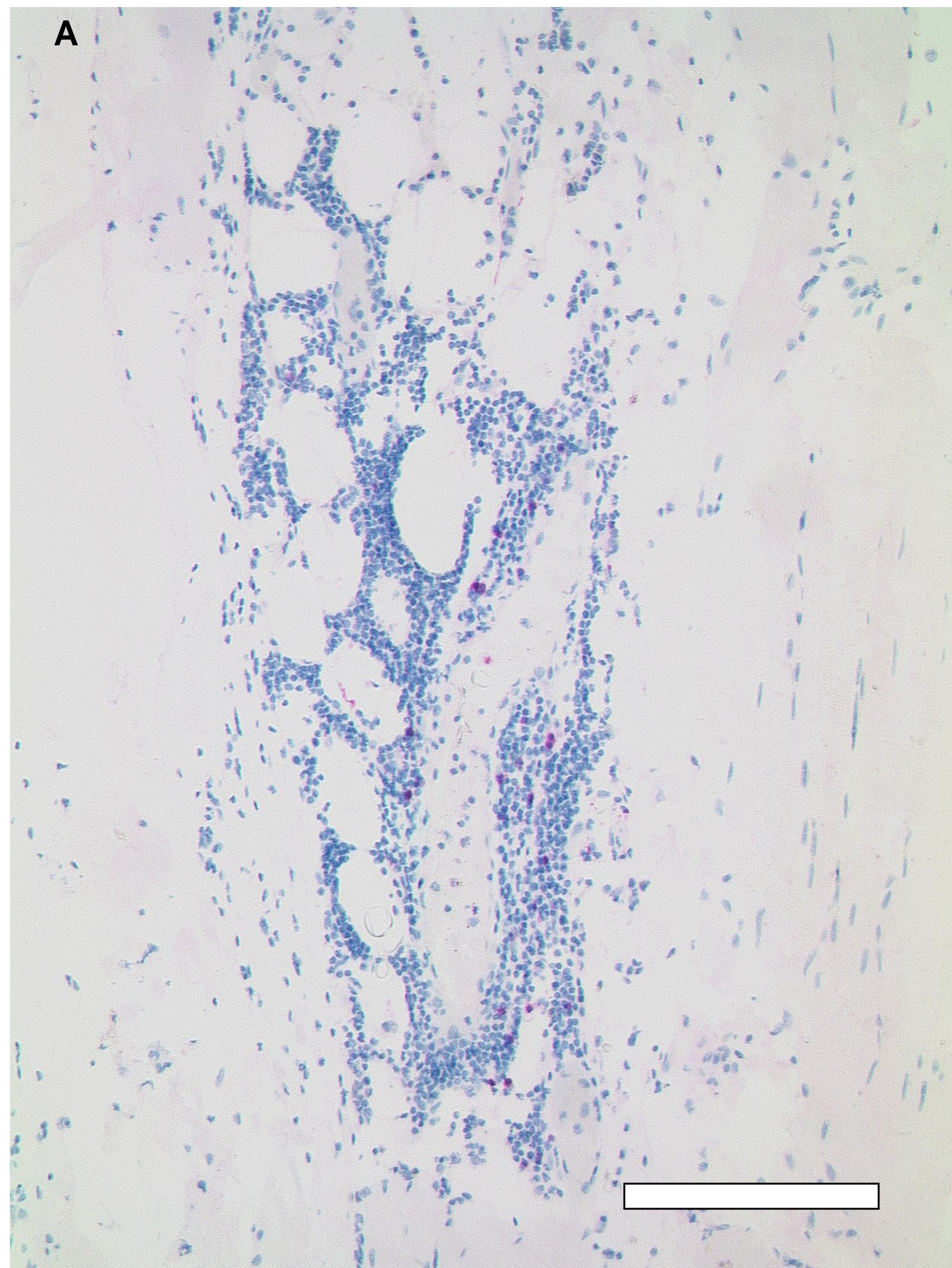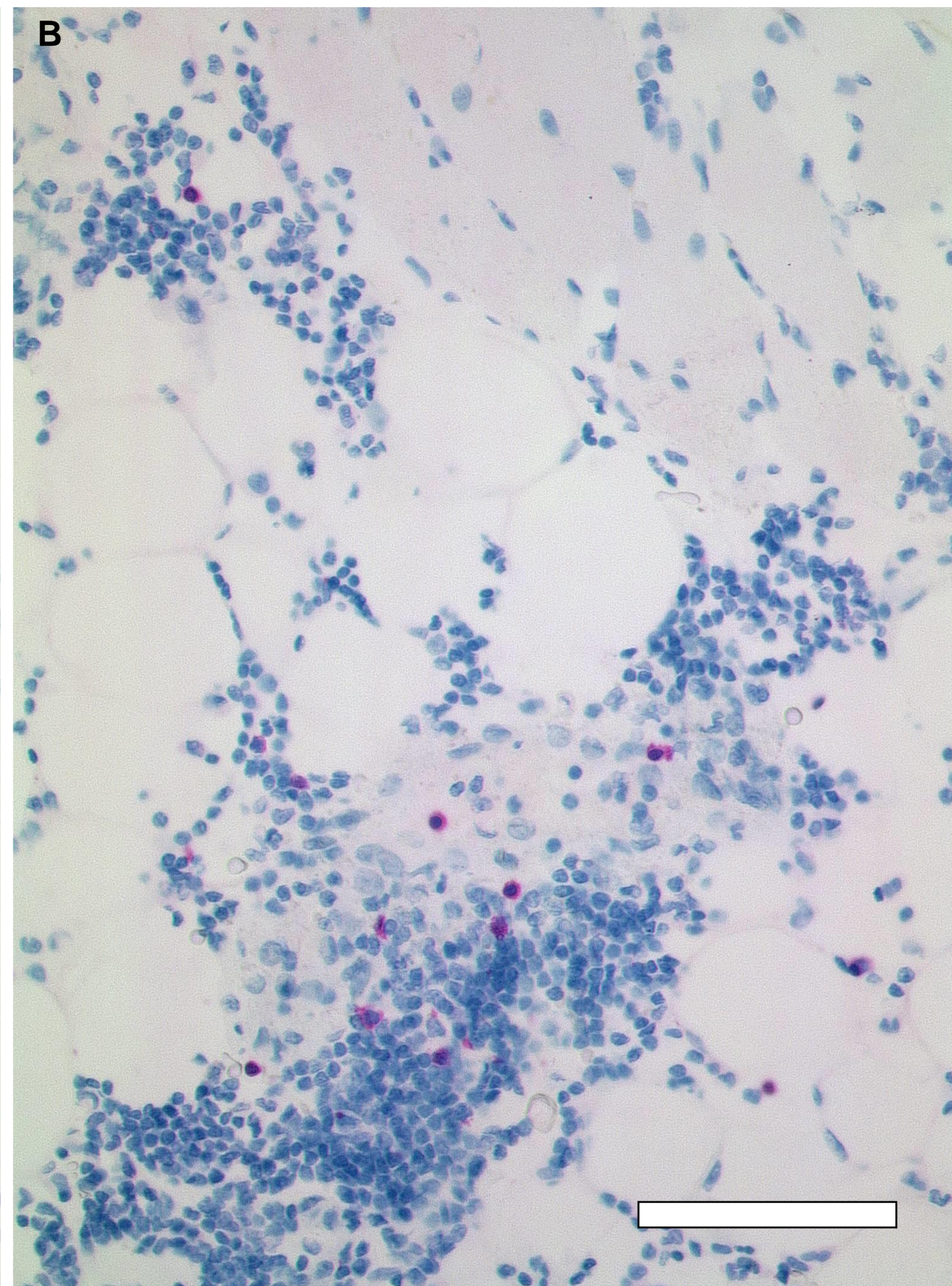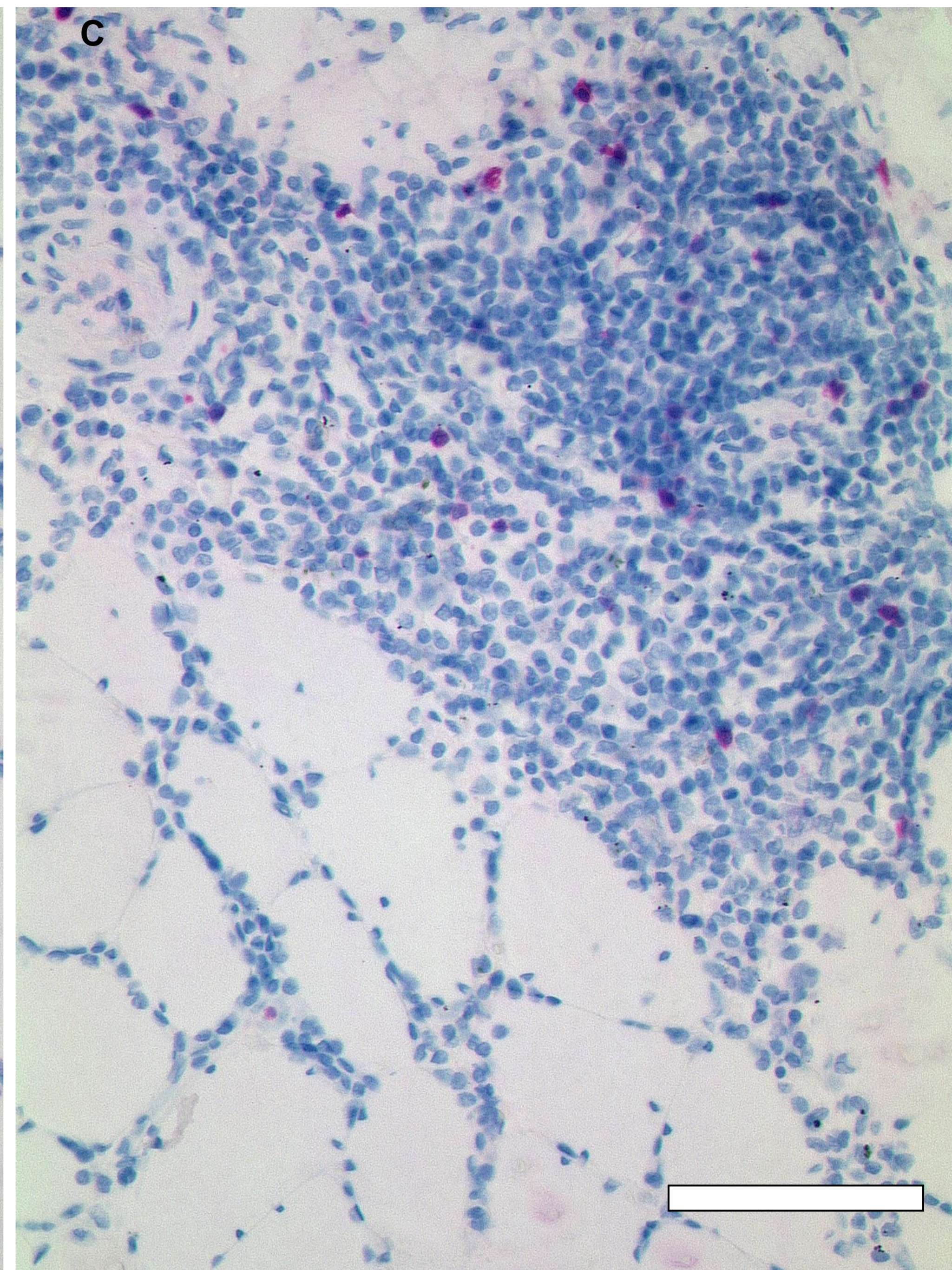

Supplement: Supplementary file 6 — Supporting information. [file IID3-11-e827-s012.pdf]

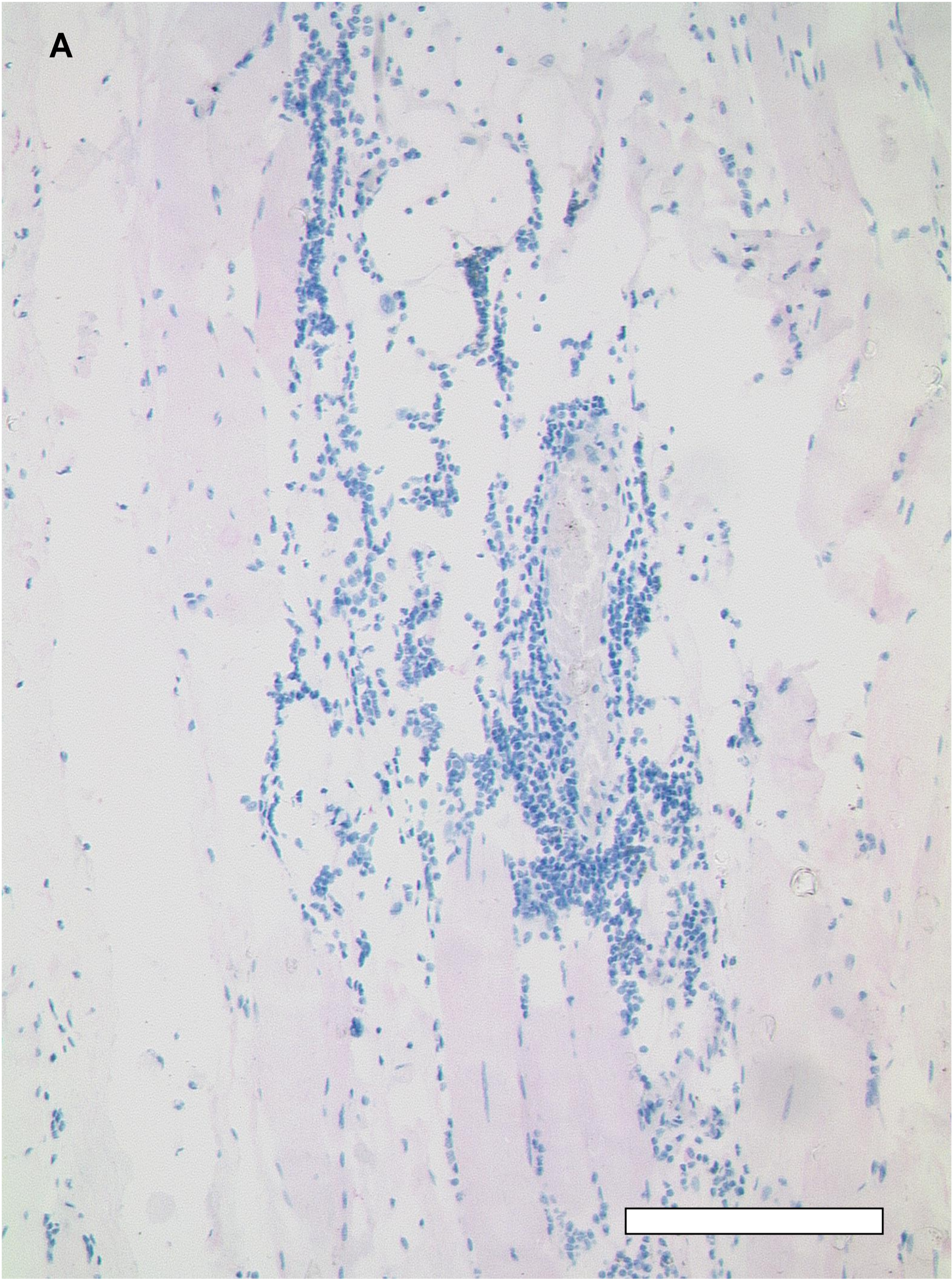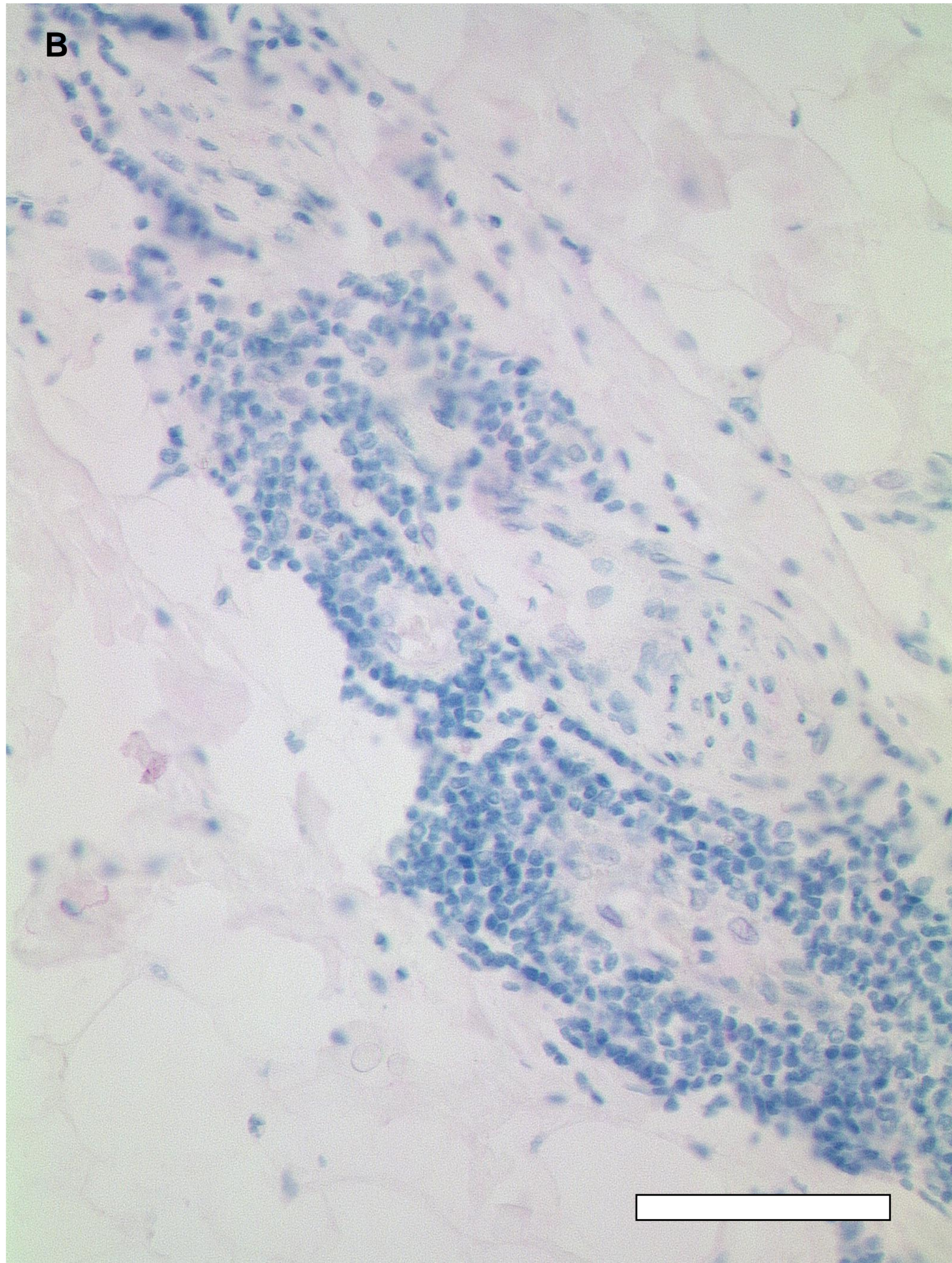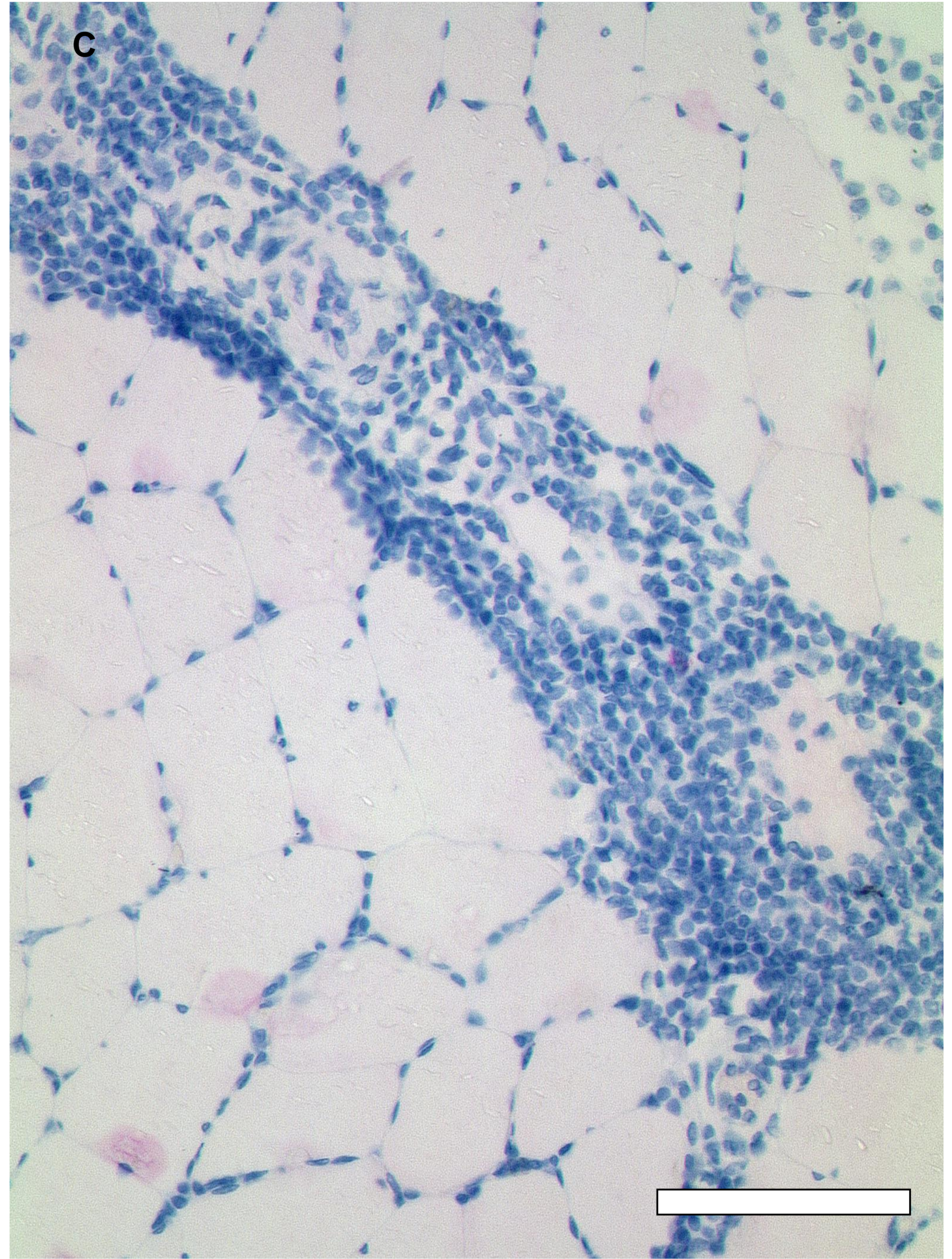

Supplement: Supplementary file 7 — Supporting information. [file IID3-11-e827-s009.pdf]

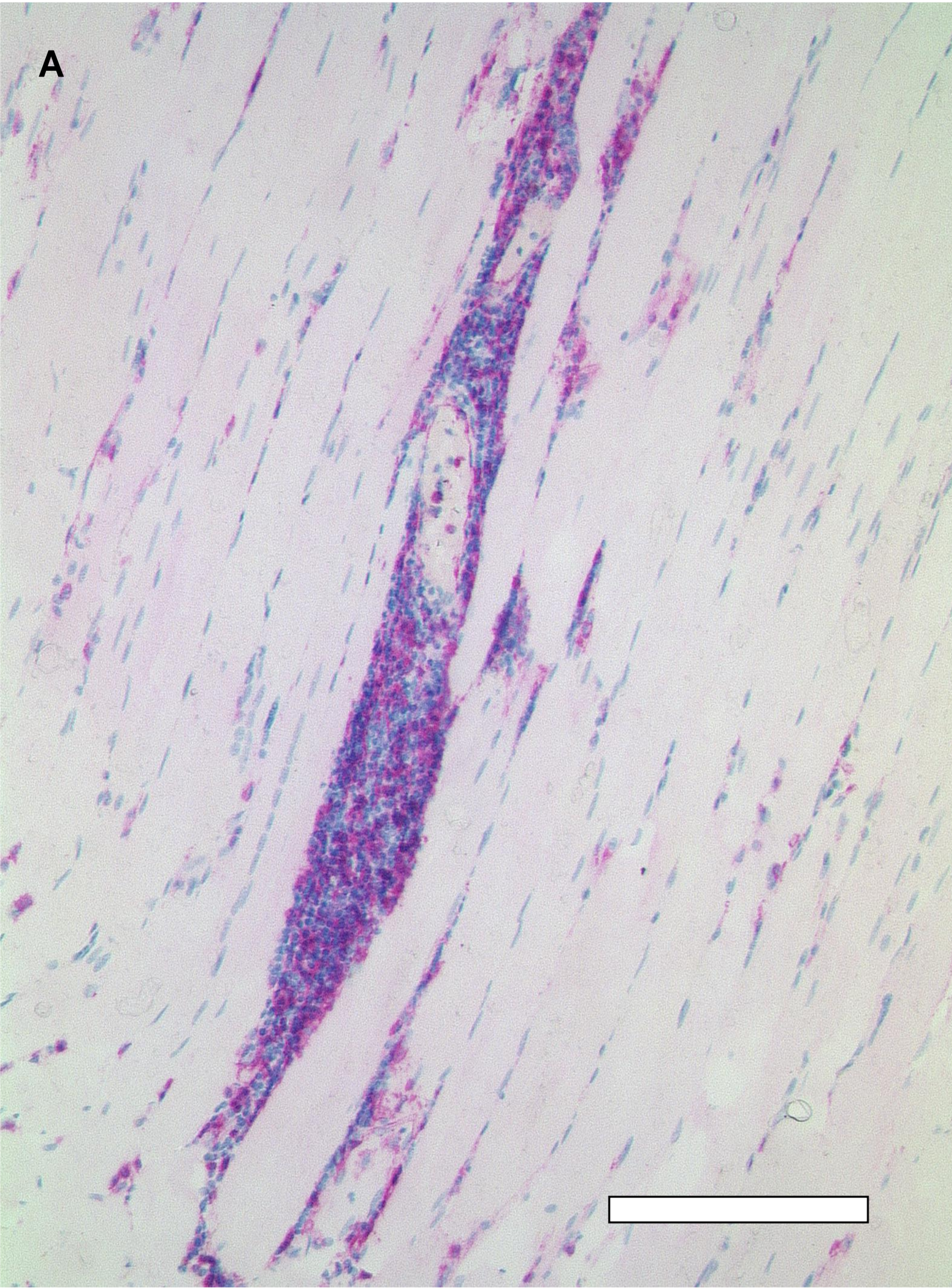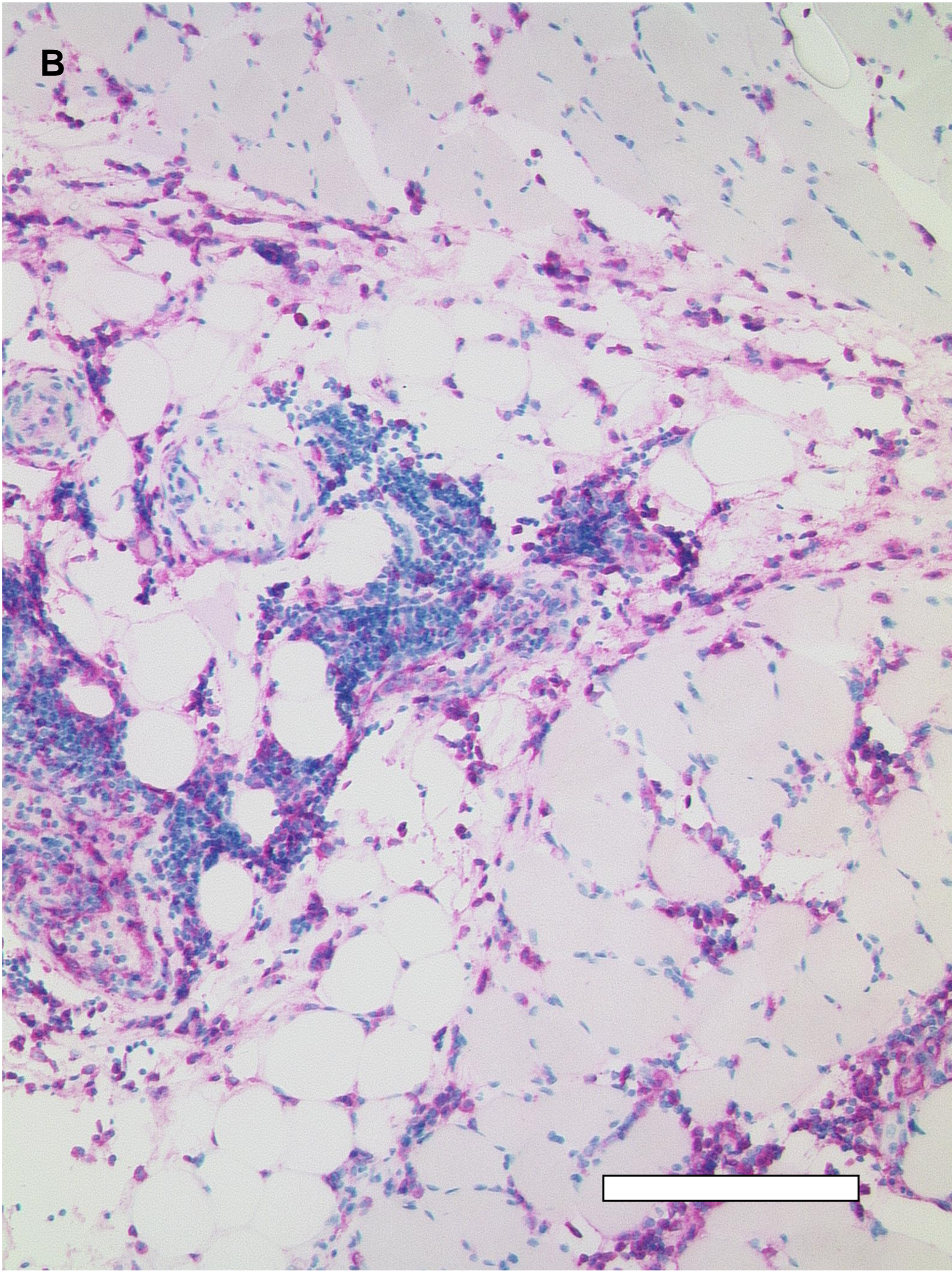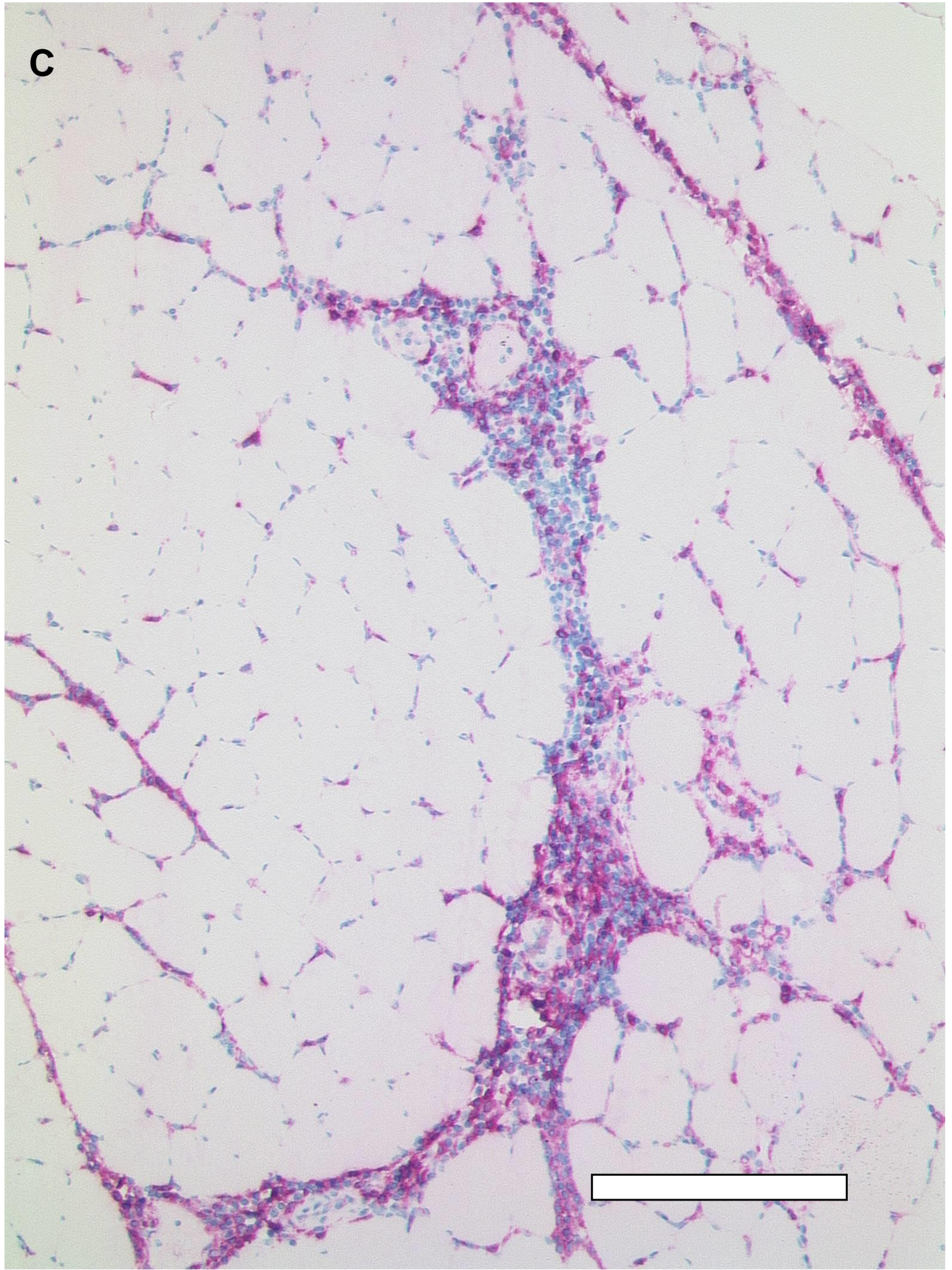

Supplement: Supplementary file 8 — Supporting information. [file IID3-11-e827-s014.pdf]

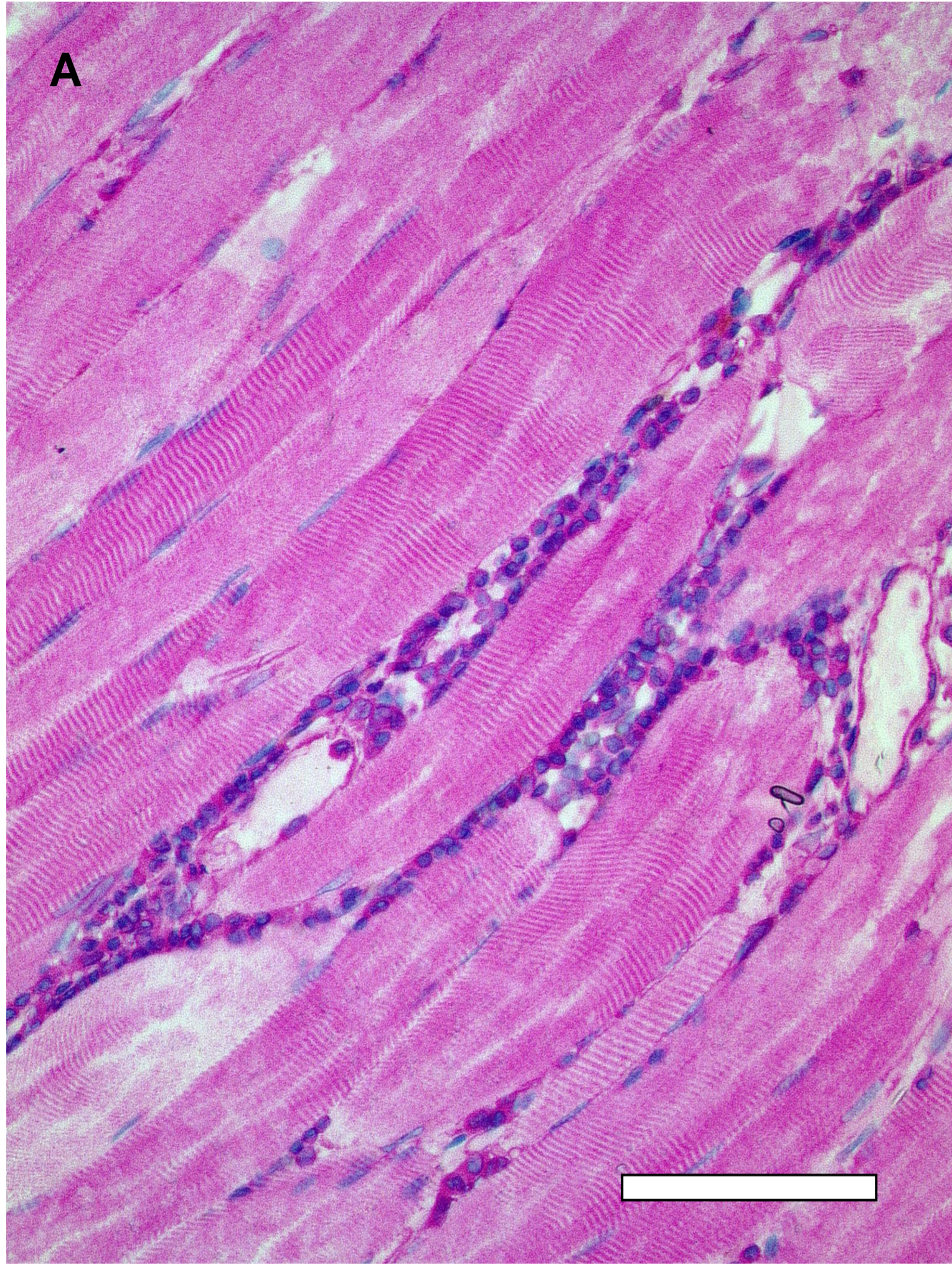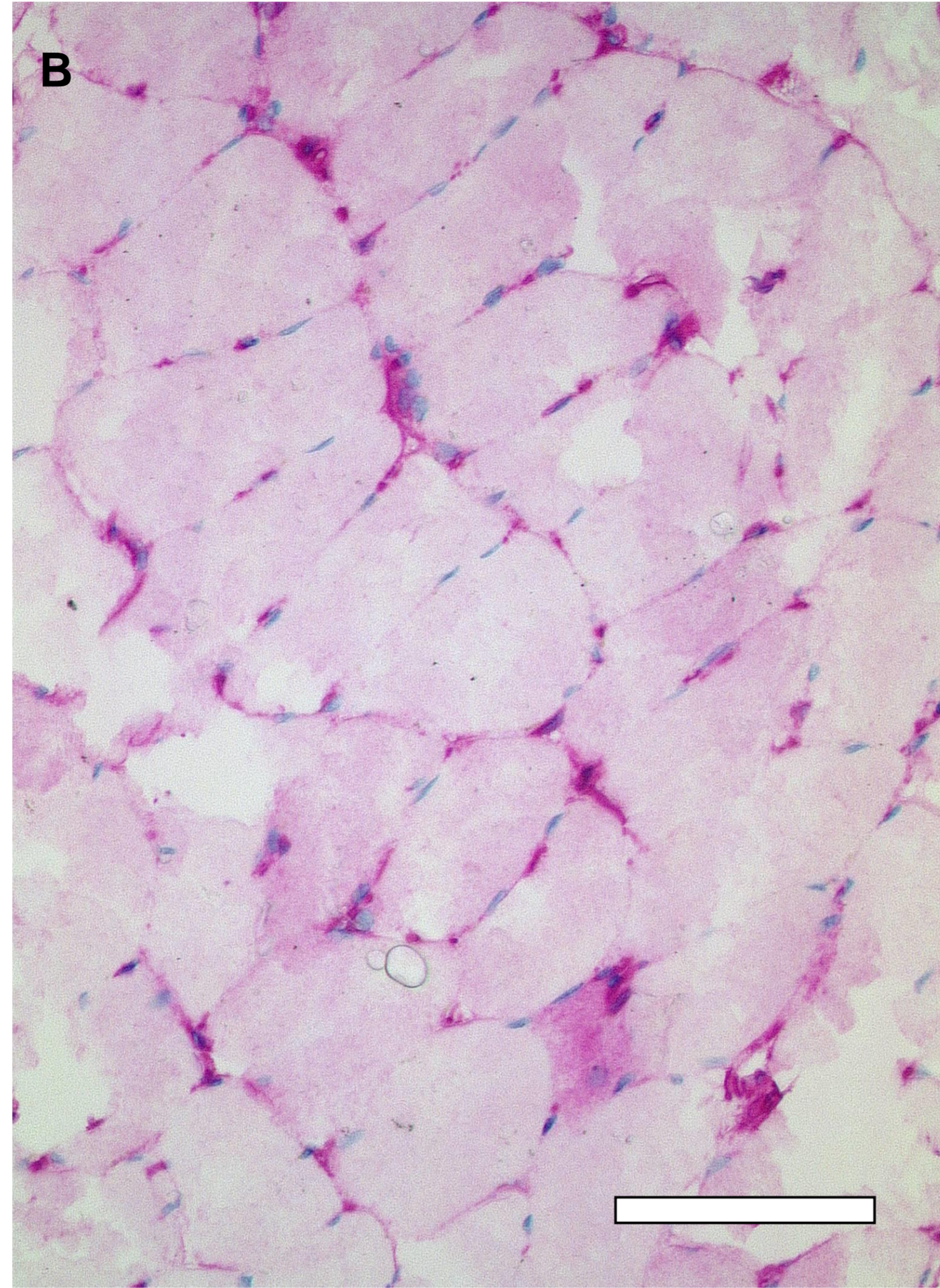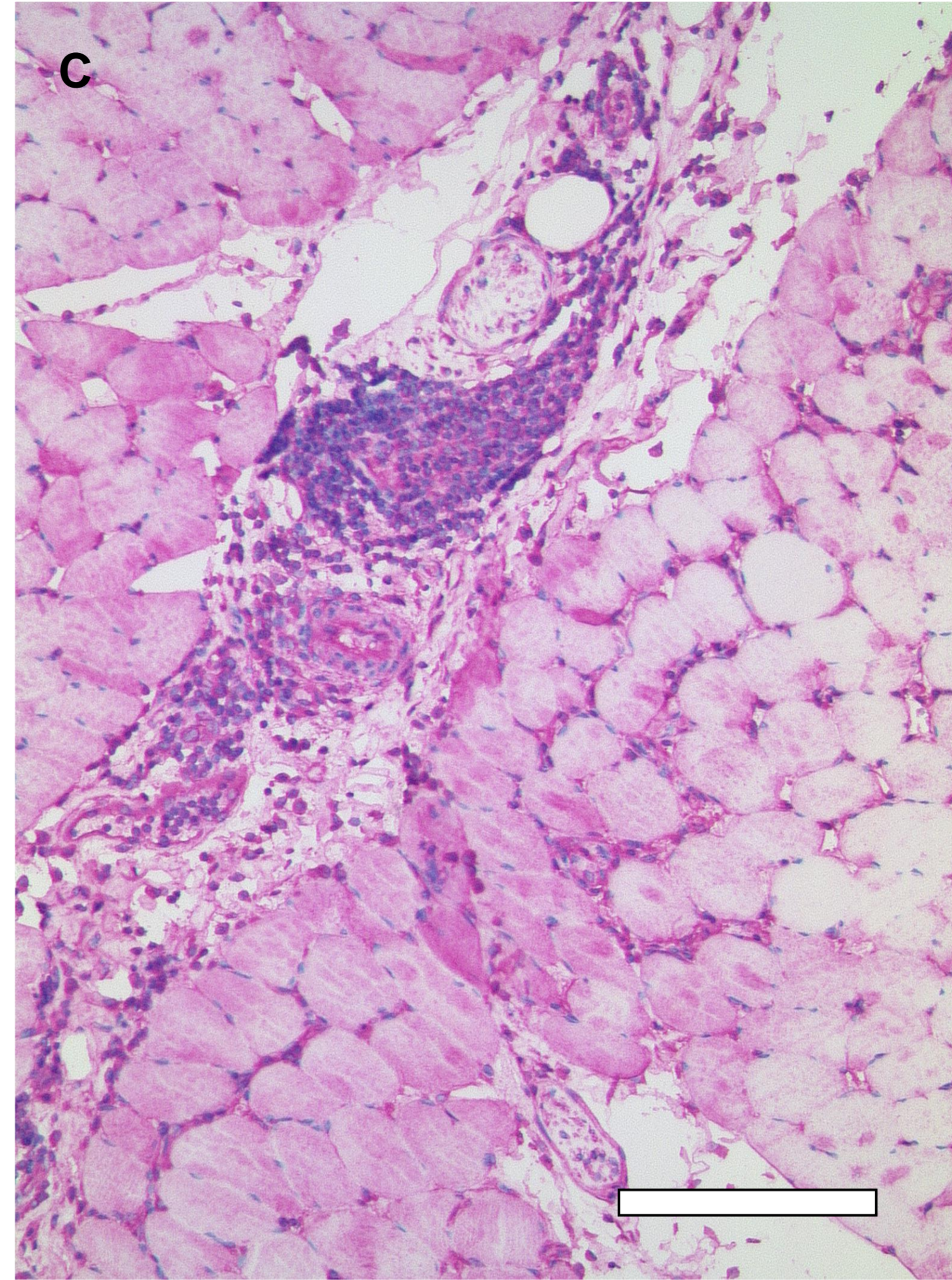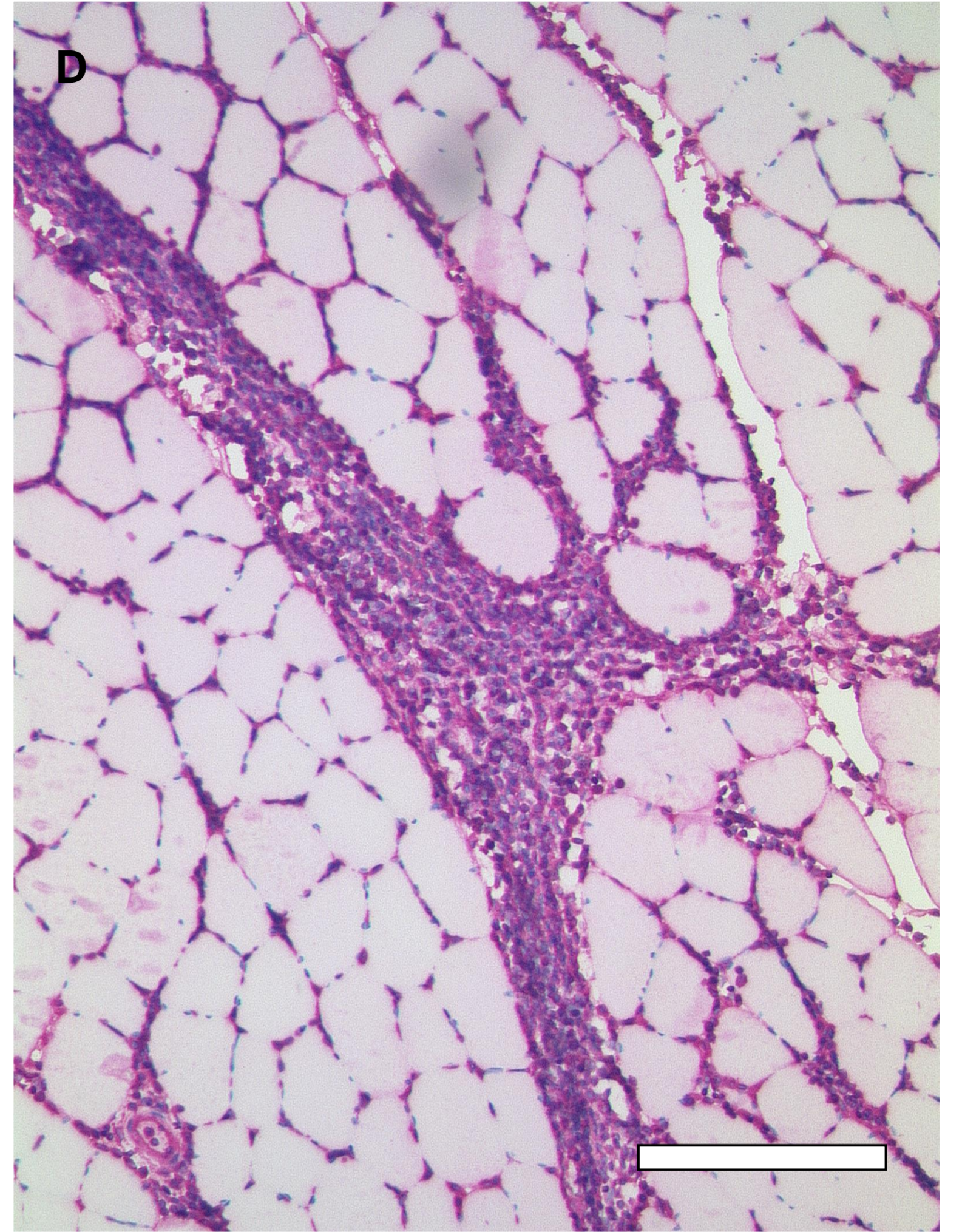

Supplement: Supplementary file 9 — Supporting information. [file IID3-11-e827-s005.pdf]

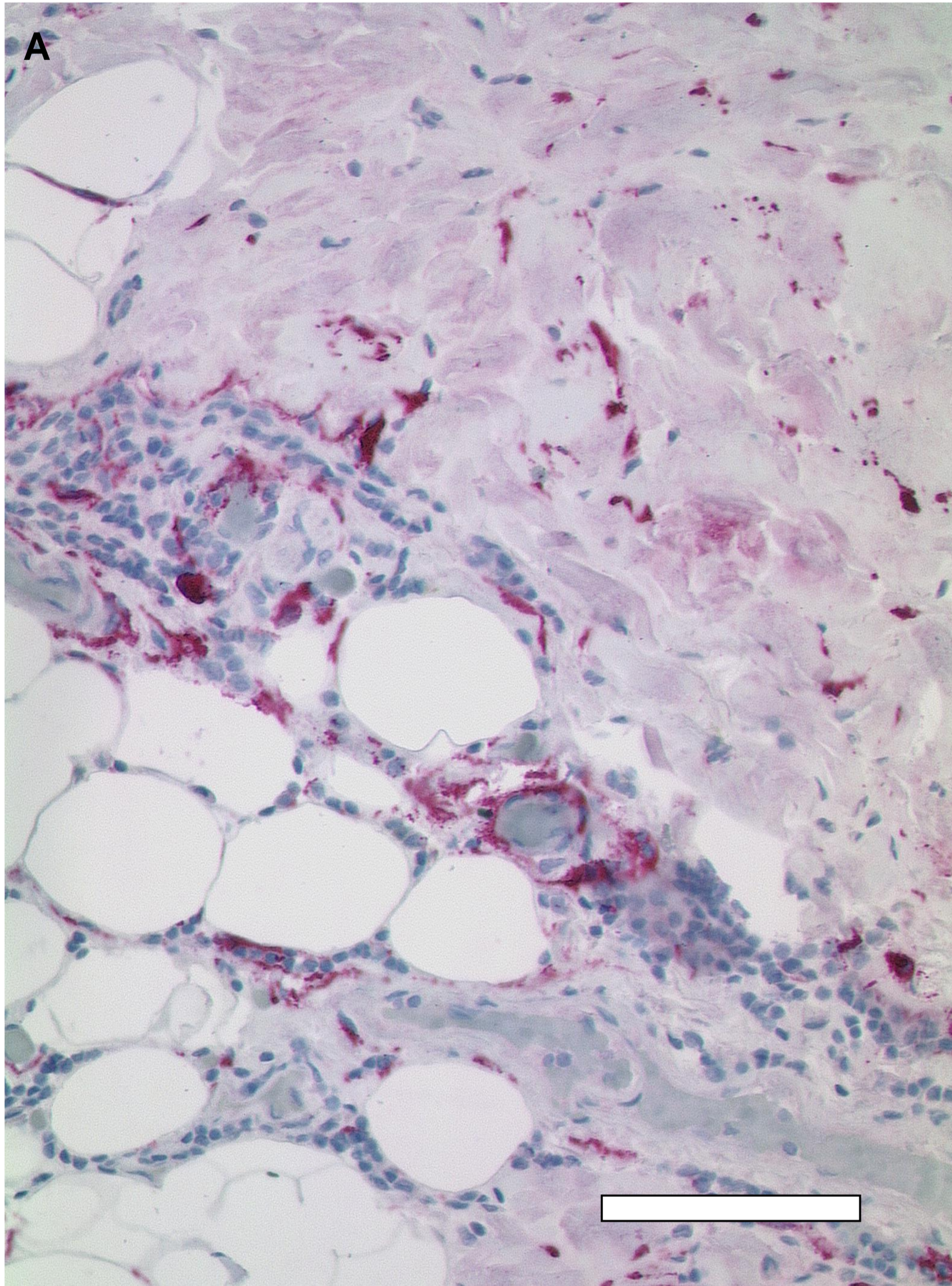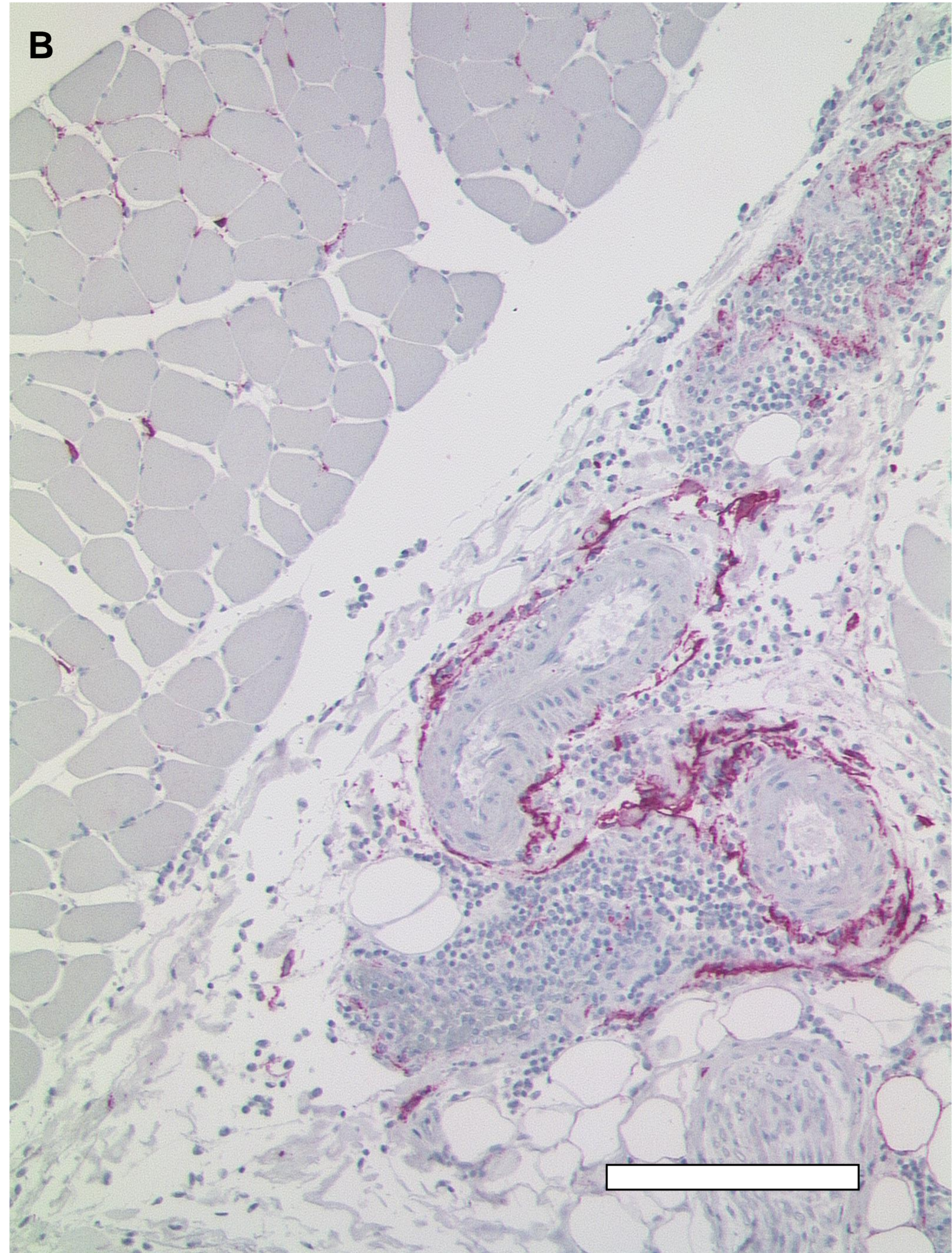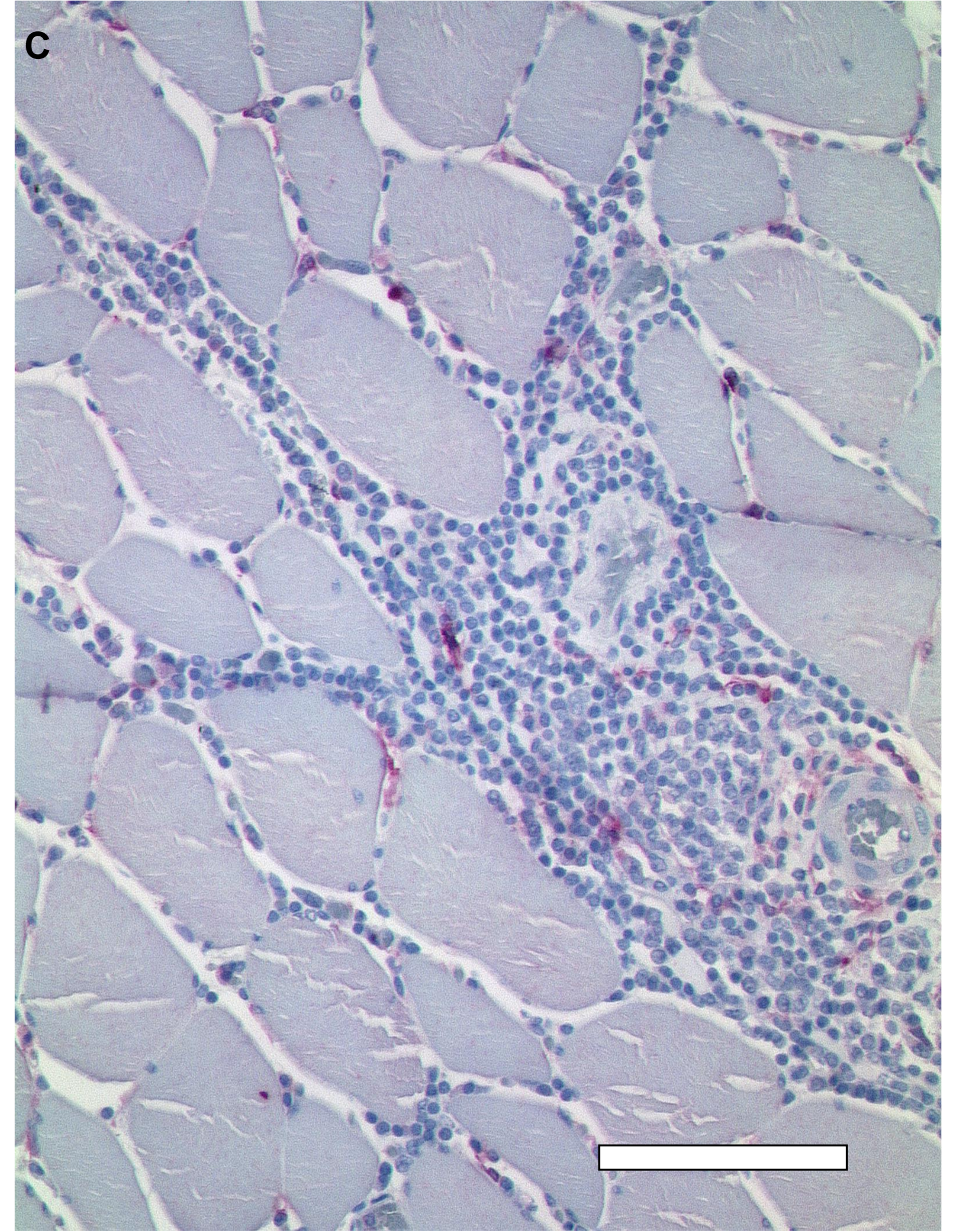

Supplement: Supplementary file 10 — Supporting information. [file IID3-11-e827-s013.pdf]

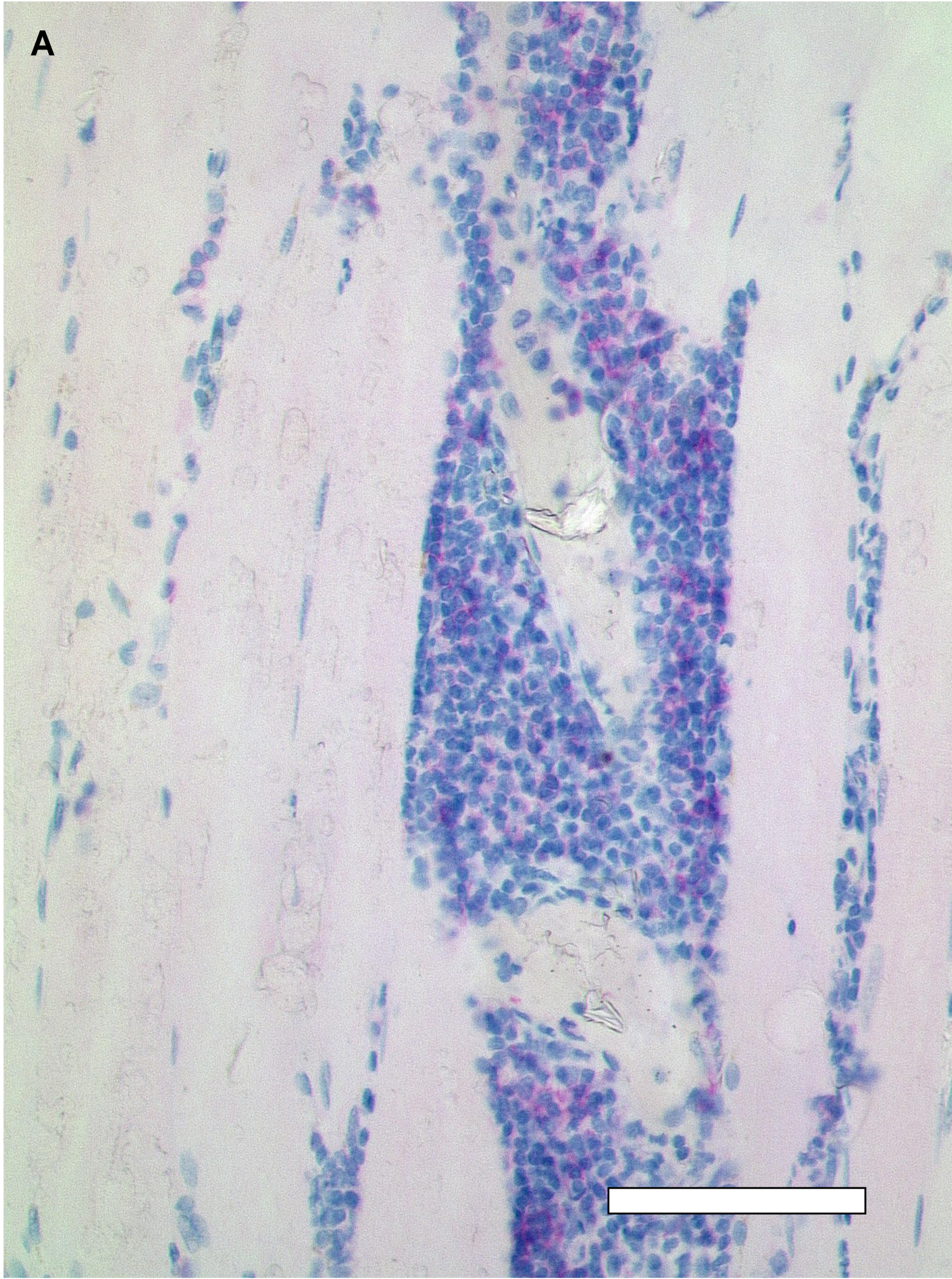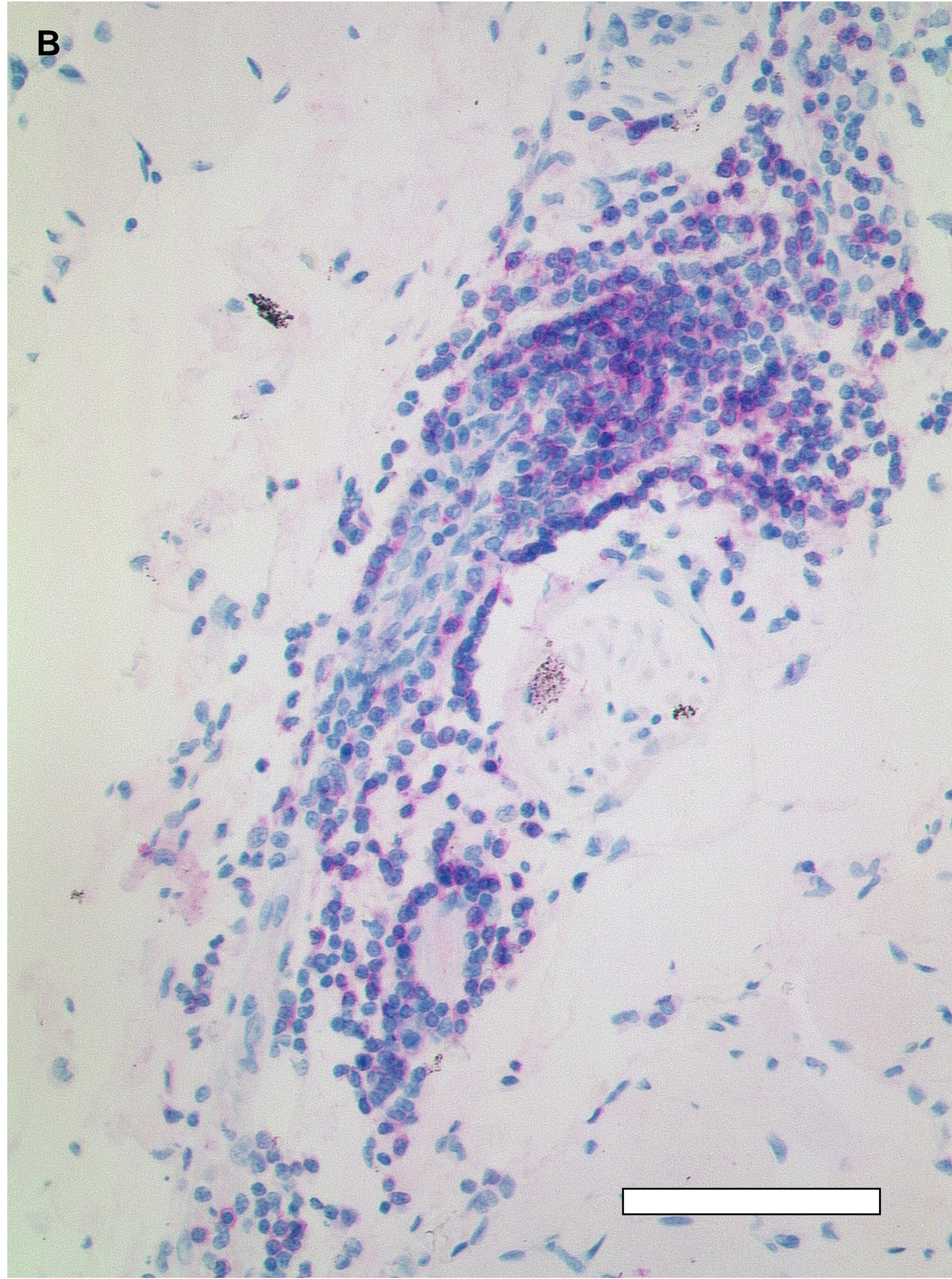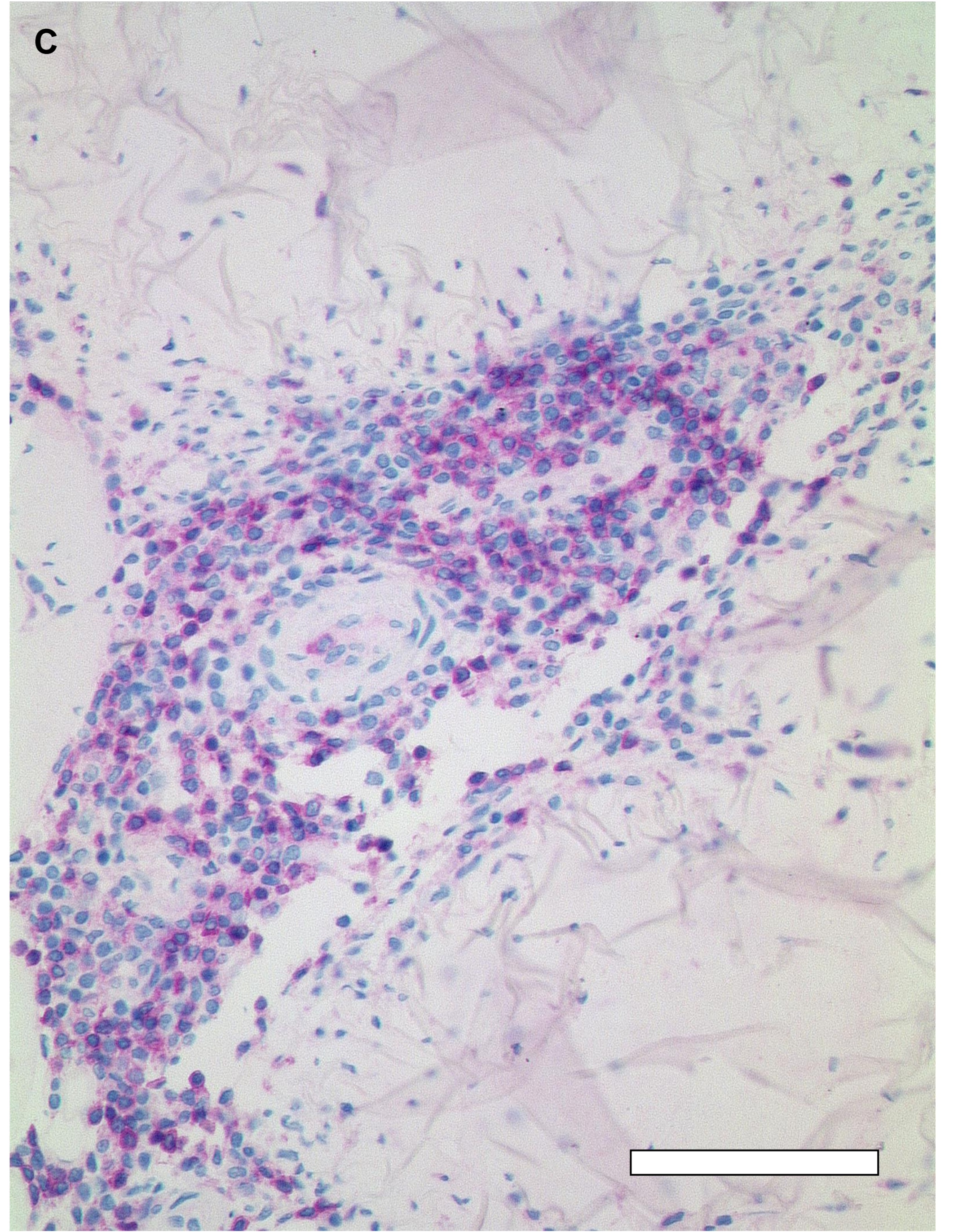

Supplement: Supplementary file 11 — Supporting information. [file IID3-11-e827-s007.pdf]

A

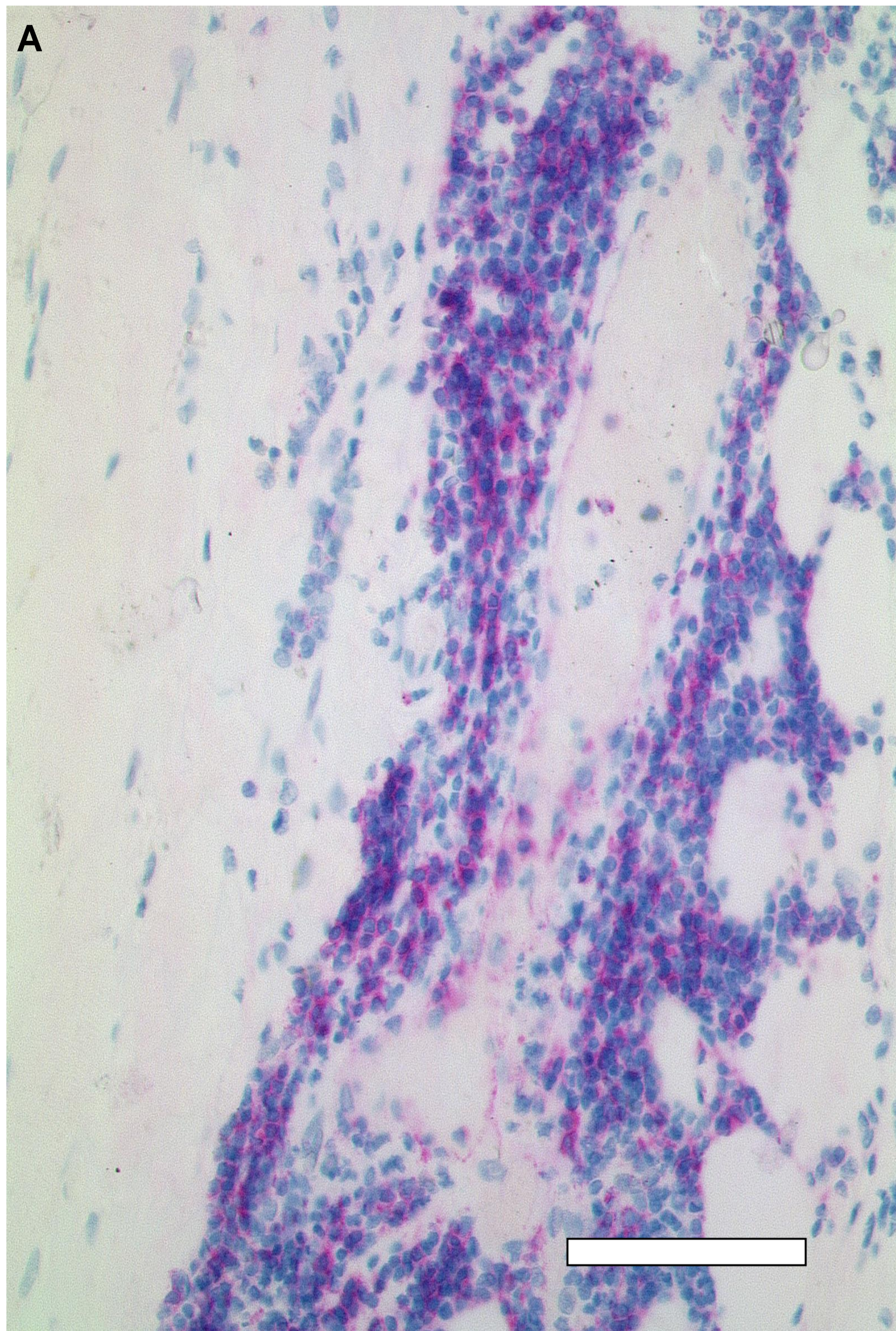

B

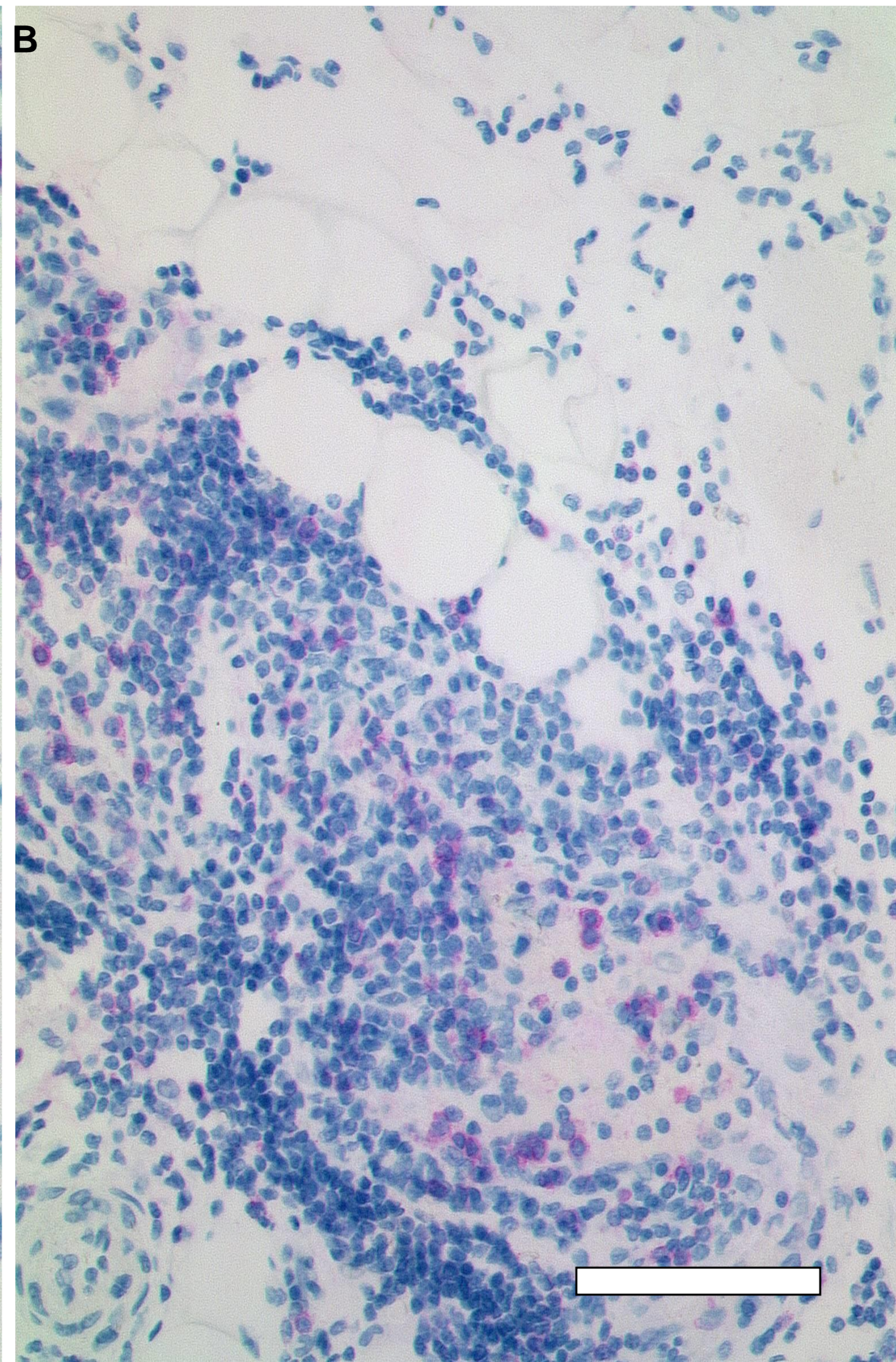

C

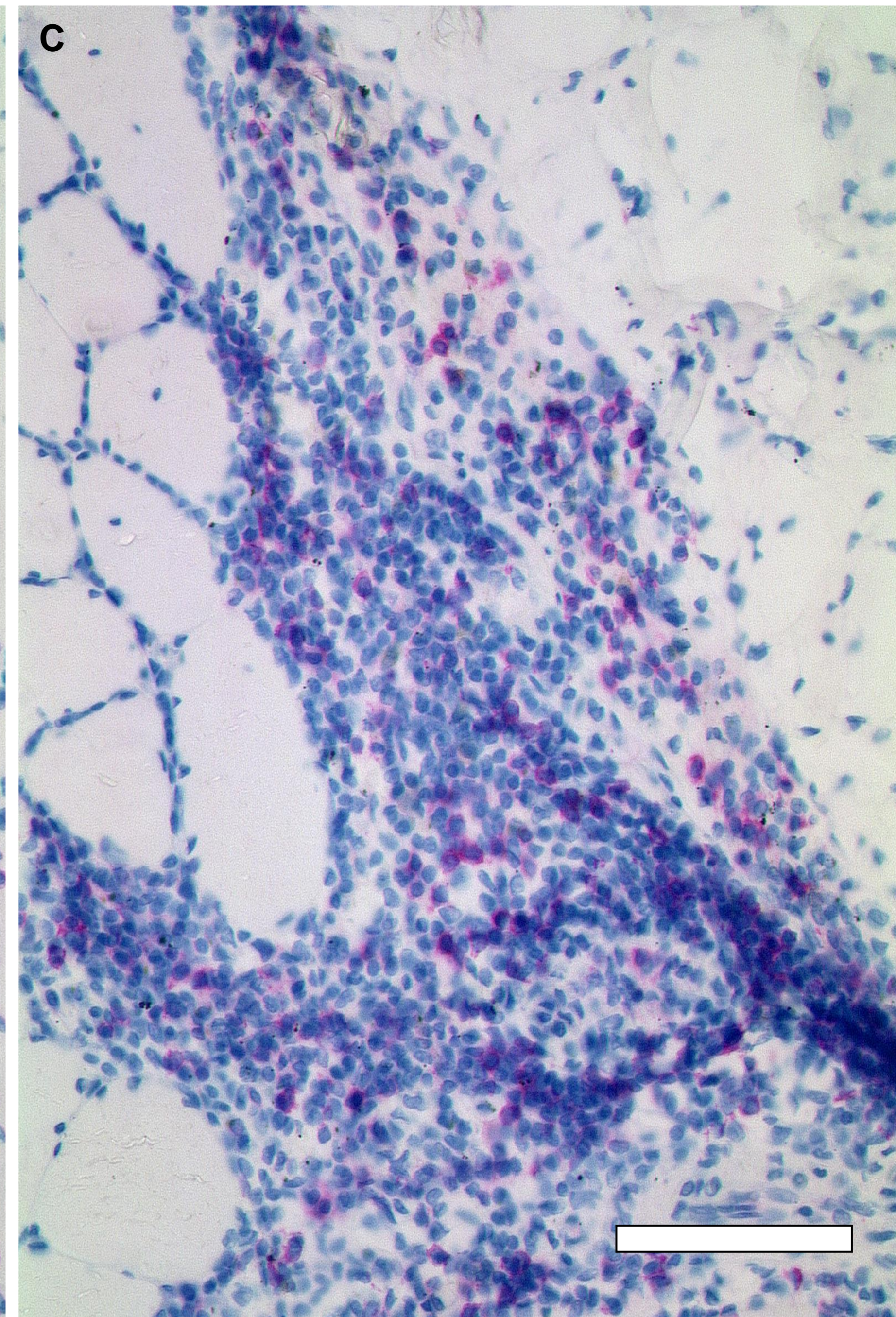

Supplement: Supplementary file 12 — Supporting information. [file IID3-11-e827-s004.pdf]

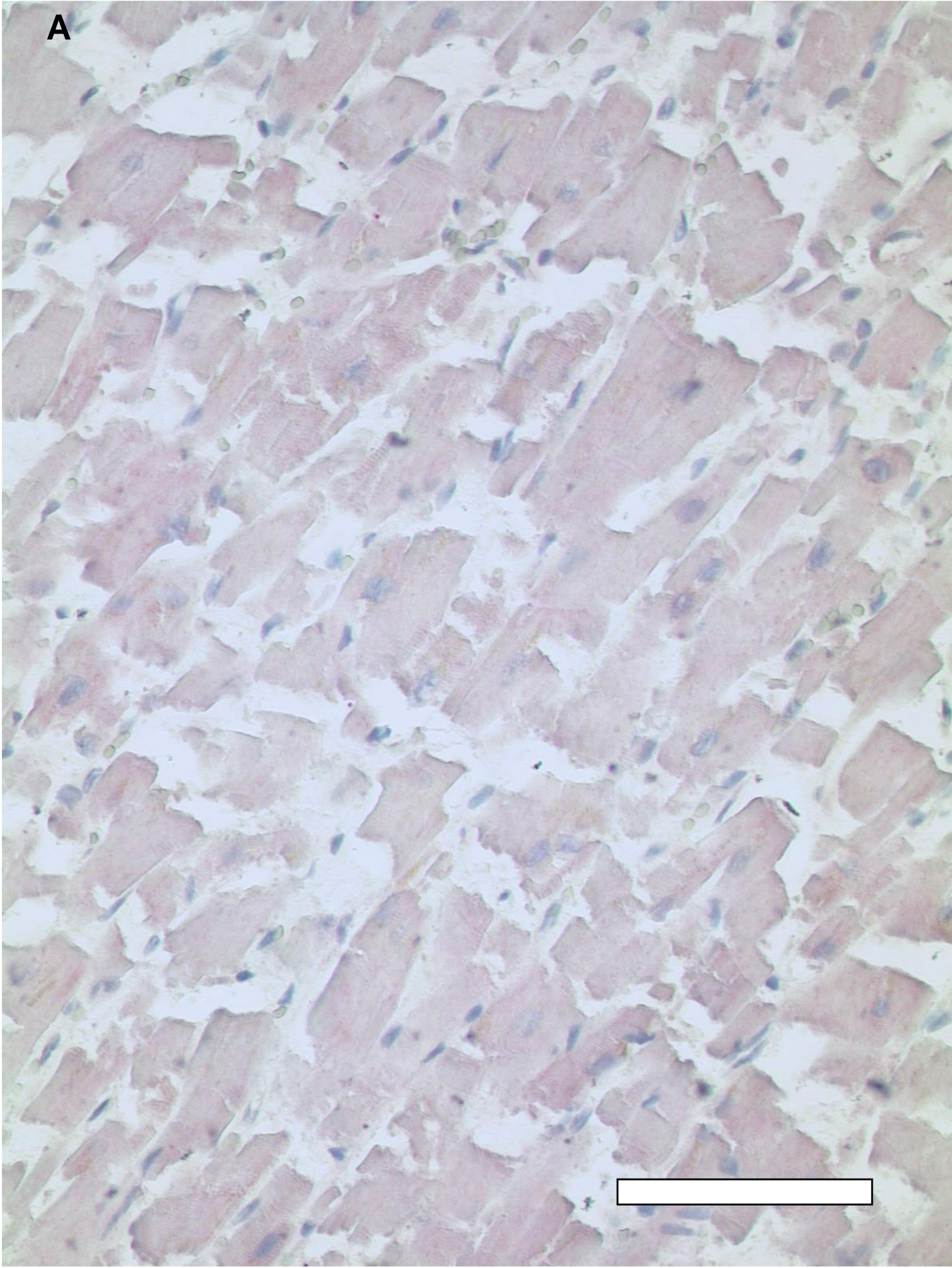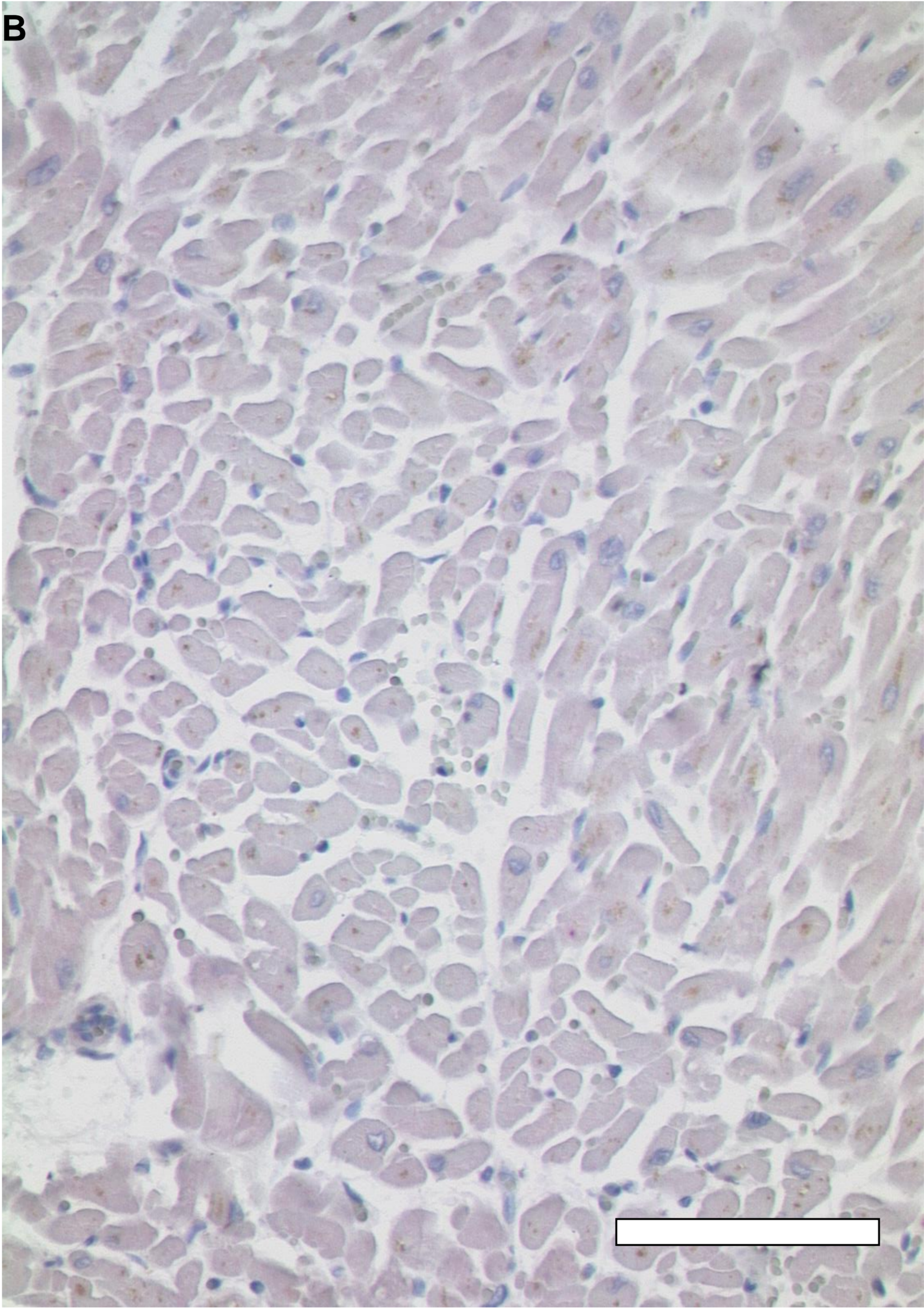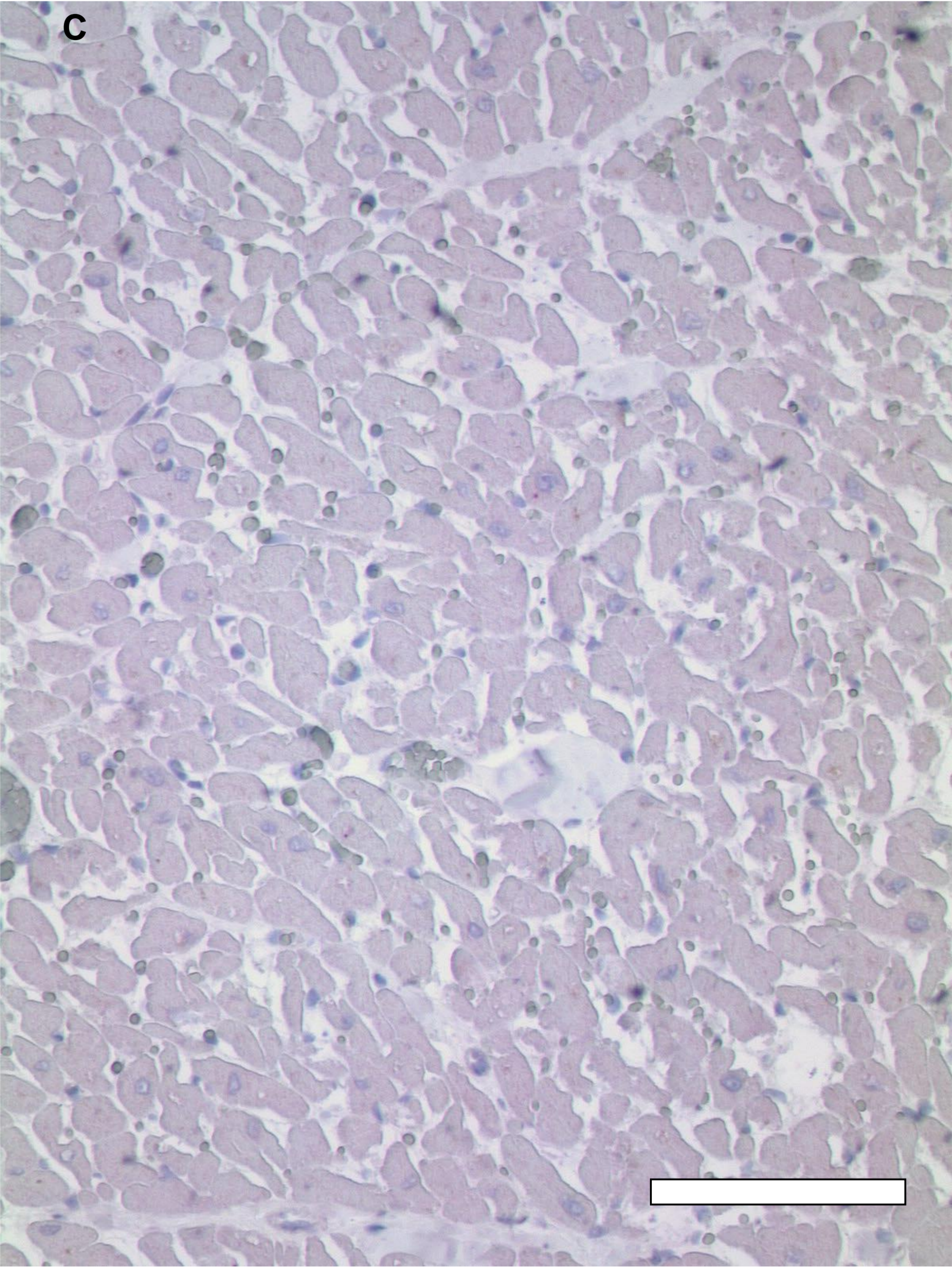

Supplement: Supplementary file 13 — Supporting information. [file IID3-11-e827-s002.pdf]
