# Supplementary material for: Emergence of SARS‐CoV‐2 spike protein at the vaccination site
Source: Immun Inflamm Dis. 2023 Mar 29;11(3):e827. doi: 10.1002/iid3.827 (PMC10052447; doi:10.1002/iid3.827)
Supplement: Supplementary file 14 — Supporting information. [file IID3-11-e827-s010.docx]

Supplemental Figure 1: Histomorphological features were similar in all three cases with a perivascular lymphohistiocytic infiltrate with extension into the muscular interstitium. ((A, Vaxzevria (case 1), focus on perivascular area, B) Vaxzevria (case 1), focus on muscular interstitium, (C) COVID-19 Vaccine Janssen (case 2), (D) Comirnaty (case 3), scale bar (A)-(D) 100 µm)

Supplemental Figure 2: In all three cases most of the lymphocytes were T-cells, positive in the CD3-stain ((A) Vaxzevria (case 1), CD3, scale bar 200 µm, (B) COVID-19 Vaccine Janssen (case 2), CD3, scale bar 200 µm, (C) Comirnaty (case 3), CD3, scale bar 100 µm)

Supplemental Figure 3: B-cells were rare in all cases ((A) Vaxzevria (case 1), CD20, scale bar 100 µm, (B) COVID-19 Vaccine Janssen (case 2), CD20, scale bar 100 µm, (C) Comirnaty (case 3), CD20, scale bar 100 µm)

Supplemental Figure 4: In all three cases CD138-positive plasma cells were virtually absent.

((A) Vaxzevria (case 1), CD138, scale bar 200 µm, (B) COVID-19 Vaccine Janssen (case 2), CD138, scale bar 200 µm, (C) Comirnaty (case 3), CD138, scale bar 100 µm)

Supplemental Figure 5: In all three cases there were lots of macrophages, especially in the periphery of the inflammatory infiltrate. ((A) Vaxzevria (case 1), CD68, scale bar 100 µm, (B) COVID-19 Vaccine Janssen (case 2), CD68, scale bar 200 µm, (C) Comirnaty (case 3), CD68, scale bar 100 µm)

Supplemental Figure 6: Only very few histiocytic cells were S100-positive ((A) Vaxzevria (case 1), S100, scale bar 200 µm, (B) COVID-19 Vaccine Janssen (case 2), S100, scale bar 100 µm, (C) Comirnaty (case 3), S100, scale bar 100 µm)

Supplemental Figure 7: Langerhans cells were not detected. ((A) Vaxzevria (case 1), CD207, scale bar 200 µm, (B) COVID-19 Vaccine Janssen (case 2), CD207, scale bar 100 µm, (C) Comirnaty (case 3), CD207, scale bar 100 µm)

Supplemental Figure 8: HLA-DR was expressed in the inflammatory cells only ((A) Vaxzevria (case 1), HLA-DR, scale bar 200 µm, (B) COVID-19 Vaccine Janssen (case 2), HLA-DR, scale bar 200 µm, (C) Comirnaty (case 3), HLA-DR, scale bar 200 µm)

Supplemental Figure 9: HLA-A,B,C was induced at different levels. The strongest induction of HLA-A,B,C was noted at the Vaxzevria injection site (case 1) ((A)HLA-A,B,C scale bar 100 µm). In contrast, the non-injected skeletal muscle showed no expression of HLA-A,B,C ((B) HLA-A,B,C scale bar 100 µm). The induction of HLA-A,B,C was mild at the injection site of COVID-19 Vaccine Janssen (case 2)((C) HLA-A,B,C, scale bar 200 µm) and not visible at the injection site of Comirnaty (case 3) ((D) HLA-A,B,C, scale bar 200 µm)

Supplemental Figure 10: Spike protein was expressed in fibrocytes and histiocytic cells with a perivascular and interstitial distribution ((A) Vaxzevria (case 1), spike, scale bar 100 µm, (B) COVID-19 Vaccine Janssen (case 2), spike, scale bar 200 µm, (C) Comirnaty (case 3), spike, scale bar 100 µm)

Supplemental Figure 11: The CD4:CD8 ratio was different in each case. The Vaxzevria injection site (case 1) contained few CD4-positive T-cells ((A) CD4, scale bar 100 µm), the COVID-19 Vaccine Janssen injection site (case 2) an intermediate number ((B) CD4, scale bar 100 µm) and the Comirnaty injection site (case 3) contained the most CD4-positive T-cells ((C), CD4, scale bar 100 µm)

Supplemental Figure 12: The Vaxzevria injection site (case 1) contained numerous CD8-positive T-cells ((A) CD8, scale bar 100 µm), the COVID-19 Vaccine Janssen injection site (case 2) ((B) CD8, scale bar 100 µm) and the Comirnaty injection site (case 3) contained only few CD8-positive T-cells ((C), CD8, scale bar 100 µm).

Supplemental Figure 13: The left ventricular myocardium did not show any expression of the spike protein. ((A) Vaxzevria (case 1), spike, scale bar 200 µm, (B) COVID-19 Vaccine Janssen (case 2), spike, scale bar 200 µm, (C) Comirnaty (case 3), spike, scale bar 200 µm).
